# Supplementary material for: Systemic cellular migration: The forces driving the directed locomotion movement of cells
Source: PNAS Nexus. 2024 Apr 20;3(5):pgae171. doi: 10.1093/pnasnexus/pgae171 (PMC11067954; doi:10.1093/pnasnexus/pgae171)
Supplement: pgae171_Supplementary_Data [file pgae171_supplementary_data.docx]

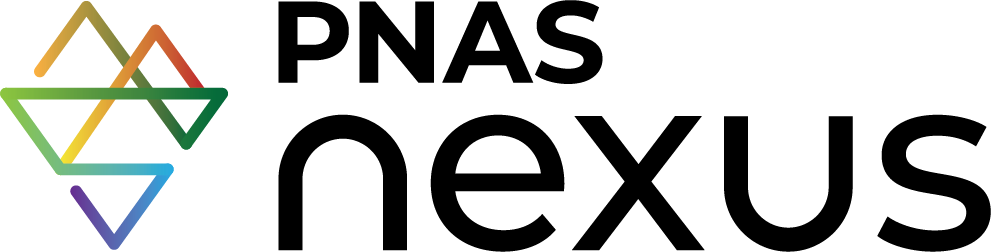


**Supplementary Information for**

Systemic cellular migration: the forces driving the directed locomotion movement of cells.

Ildefonso M. De la Fuente^1,2^, Jose Carrasco-Pujante^3^, Borja Camino-Pontes^4^, Maria Fedetz^5^, Carlos Bringas^3^, Alberto Pérez-Samartín^6^ Gorka Pérez-Yarza^3^, José I. López^4^, Iker Malaina^1*^, Jesus M Cortes^3,4,7*^

Ildefonso Martínez De la Fuente

Email: mtpmadei@ehu.eus

ildefonso@cebas.csic.es

**This PDF file includes:**

Supplementary text

Figures S1 to S4

Tables S1 to S10

SI References

**Materials and Methods**

Cell cultures

The three species of free-living amoebae were cultured in Ø100 x 20 mm Petri dishes (Corning^®^ CLS430167) at 21ºC. *Amoeba proteus* (Carolina Biological Supply, #131306) and *Metamoeba leningradensis* (CCAP: Culture Collection of Algae and Protozoa, Oban, Scotland, United Kingdom, catalogue number 1503/6) were grown in Chalkley’s simplified medium (1) (NaCl, 1.4 mM; KCl, 0.026 mM; CaCl2, 0.01 mM) with heat-treated wheat grains. *Amoeba borokensis* (ACCIC (2): Amoebae Cultures Collection of Institute of Cytology in Saint Petersburg) were grown in Prescott & Carrier media (3) and fed every fourth day 0.5 ml of a mixed *Chilomonas sp.* (Carolina Biological Supply^®^ #131734) and *Colpydium sp.* (ACCIC culture. *Chilomonas sp.* and *Colpydium sp.* were grown in Prescott & Carrier media as well.

Experimental set-up

A schematic drawing of the experimental set-up is shown in Fig. 1. Two electrophoresis blocks (BIO-RAD Mini-Sub cell GT) were interconnected by two ~12 cm long agar bridges (2% agar in 0.5 N KCl in) and the first one was plugged into a BIO-RAD Model 1000/500 power supply unit. Atop the central elevated platform of the second block, a custom-devised experimental glass chamber was placed. By using agar bridges and removing all the electrodes from the second electrophoresis block we avoided direct contact between the media, where the cells moved, and any metallic electrodes, thus preventing ionic contamination.

The experimental glass chamber consisted of a modified 25 x 75 x 1 mm standard glass slide and three additional, smaller glass pieces (one central piece measuring 24 × 3 × 0.17 mm and two flanking pieces of 24 × 40 × 0.17 mm each) crafted by carefully trimming three 24 x 60 x 0.17 mm cover glasses (Fig. 1). To build the modified standard glass slide, two 24 x 60 x 0.1 mm cover glasses were stuck using silicone to a standard glass slide and let dry for 24h, whereupon the 20 × 60 × 0.17 mm overhanging parts from both cover glasses were cut off, leaving two 4 × 60 × 0.17 mm glass sheets as the chamber’s sideway walls. All the experiments were conducted within the experimental glass chamber, which allowed to establish a laminar flow when closed and to place or extract the cells when opened.

Fresh agar bridges were used in every experimental replica to avoid contamination and conductivity loss (leak of KCL into the simplified Chalkley’s medium, which has a much lower osmotic concentration than the agar bridges). Furthermore, one electrophoresis block was used exclusively for chemotaxis and another one for galvanotaxis, while the whole set-up (both the electrophoresis blocks and the glass chamber) was scrupulously cleaned and reassembled after each experimental replica.

Ahead of every experiment, the modified glass slide was adhered to the top of the central platform of the second electrophoresis block using a droplet of olive oil to prevent both the medium and the electric current from passing beneath the experimental glass chamber. Following this, the 24 × 3 × 0.17 mm central glass piece was gently placed atop the middle of the modified glass slide. Next, amoebae were washed in fresh simplified Chalkley’s medium and placed beneath the central piece of the glass chamber, where they were left to attach to the surface of the modified glass slide for ~2 minutes. This step needs to be performed in less than 15 seconds, for the amoebae will immediately start attaching to the inner surface of the plastic micropipette tip, and any further pipetting will henceforth potentially damage their cellular membrane. *Metamoeba leningradensis* cells attached faster and stronger to the plastic micropipette tips than the other two species, thus being especially susceptible to being damaged. Subsequently, the two 24 × 40 × 0.17 mm lateral sliding glasses were laid flanking the central piece and slightly overhanging the electrophoresis block wells. Each well was carefully filled with 75 ml of clean simplified Chalkley’s medium, and the two 24 × 40 × 0.1 mm lateral sliding glasses were gently poked down using a micropipette tip until they contacted the medium, which spread beneath them by surface tension. Finally, the two lateral glass pieces were longitudinally slid till they touched the central piece where the amoebae laid, thus closing the glass chamber, and establishing a connection between the media in both wells.

All experimental replicates were performed with a maximum 9 cells and lasted for exactly 30 minutes, during which cell behavior was recorded using a digital camera. In preparation for each experiment, amoebae were starved for 24 hours in fresh, nonnutritive simplified Chalkley’s medium. Only cells deemed healthy (motile and rod-shaped) were chosen for the experiments. Deviations from optimal culture and experimental conditions, as well as mechanical issues in the recording system, rendered ⪅ 9% of the experimental replicates invalid. Those replicates have not been considered in this investigation.

Galvanotactic stimulus

The galvanotactic stimulus is traumatic for the amoebae, and thus can affect their response. Four key steps were taken to minimize those effects by ensuring optimal current intensity and voltage in all the experiments where a galvanotactic stimulus was applied: 1. The power supply unit was programmed to keep the voltage at 60V; 2. The flow sectional area of the experimental glass chamber was adjusted by modifying the amount of silicone used to glue the longitudinal walls of the modified glass slide, which determined their height; 3. A variable 1 MΩ resistor and a microammeter were installed in series, in that order (Fig. S1), and the intensity of the electric current was manually corrected when needed in real time by turning the variable resistor’s screw to tune the global resistance of the set-up; thus, a stable 60V electric potential and optimal current intensity values of 70-74 µA (*A. proteus*), 70-80 µA (*M. leningradensis*) and 68-75 µA (*A. borokensis*) were kept throughout the galvanotactic experiments; 4. Lastly, the current was stopped immediately once the 30-minute image recording finished.

We found some amoebae populations displayed an anomalous, inverted or even null response to the galvanotactic stimulus. We therefore carried out a 5-minute galvanotactic test prior to any experiments where amoebae were exposed to galvanotaxis for the first time, using intensity and voltage values within the optimal ranges provided.

Chemotactic stimulus & gradient calculation

To establish the chemotactic peptide gradient, we added 750 µl of 2 × 10^−4^ M nFMLP (Sigma-Aldrich, #F3506) to one well of the second electrophoresis block to obtain a working peptide concentration of 2 × 10^−6^ M. The medium in that well was stirred immediately after adding the peptide to properly mix the peptide until the amoebae began to respond to it.

To assess the nFMLP peptide gradient concentration, an experiment was carried where 60 µL of medium were sampled at 0, 2, 5, 10, 15, 20, and 30 minutes since the addition of the nFMLP peptide. Samples were taken from the center of the experimental glass chamber through a small gap opened between the central glass piece and one of its flanking glass pieces by slightly moving this sliding lateral glass. Known fluorescein-tagged peptide concentration values from a standard curve were used to extrapolate nFMLP concentration at each time point (Fig. S2). Two samples were taken at each time point, and the whole procedure was replicated three times, yielding a total of 6 measurements for each time point. Fluorescence was measured at 460/528 excitation/emission wavelengths on 96 well glass bottom black plates (Cellvis, #P96-1.5H-N) using a SynergyHTX plate reader (BIOTEK) following the protocol established by Green and Sambrook (4).

Experiment recording and cell tracking

Experiments were recorded with a digital camera attached to an SM-2T stereomicroscope. Two frames were captured every second for 30 minutes (3600 frames). Individual cell movements (tracks) were manually digitized using the TrackMate (5) plugin from FIJI (ImageJ) and saved as a list of (x, y) coordinate tuples. Manual tracking was chosen over automated and semi-automated alternatives due to the well-known imprecision of such tools (6).

Root mean square fluctuation (rmsf) analysis

The root-mean-square fluctuation (rmsf) analysis is a traditional approach in Statistical Mechanics built upon the concepts introduced by Gibbs in 1902 (7) and Einstein in 1909 (8). It was later expanded and used to measure physiological signals (as per 9,10). We employed the rmsf method to evaluate the existence of power-law autocorrelation in the time-series of move-step fluctuations, adhering to the procedure outlined by Viswanathan et al. in 1996 (11). To calculate the rmsf in a given two-dimensional trajectory $P(t)=[x(t), y(t)]$ with values equidistant in time, we first defined the displacement time series as: $u\left( t \right)=\sqrt{\left[ y\left( t+1 \right)-y\left( t \right) \right]^{2}+\left[ x\left( t+1 \right)-x\left( t \right) \right]^{2}}$, for $t =1,..., t_{max}$. From here, the net displacement of the time series after $l$move-steps was computed as $y\left( l \right)\equiv\sum_{i=1}^{l} u(i)$. Finally, the rmsf of the average displacement is calculated as $F(l)\equiv\sqrt{<\Delta y\left( l \right)^{2}>-<\Delta y\left( l \right)>^{2}}$, where we have defined $\Delta y\left( l \right)\equiv y\left( l+l_{0} \right)-y\left( l_{0} \right)$ and the brackets denote the average over all possible values of *l_0_*. Therefore, *F*(*l*) is defined as the square root of the difference between the average of the square of Δ*y*(*l*) minus the square of its average. Persistence can be identified when the fluctuations follow a power law, i.e., $F(l)\sim l^{\alpha}$. For uncorrelated data, the fluctuation exponent $\alpha$ is equal to 0.5, whereas a value between 0.5 and 1 signifies the presence of positive persistence (11).

Mean Square Displacement

The Mean Squared Displacement (MSD) is a technique that was first introduced by Einstein in his studies on Brownian motion (12). Since then, it has been extensively used in various fields, including the quantification of cell motility (13, 14). This method calculates the average squared displacement in a migration path over progressively increasing time scales (14). In essence, the MSD serves as an indicator of the area traversed by the cell over time and is associated with the overall efficiency of migration (15). For a two-dimensional trajectory $P\left( t \right)=[x\left( t \right), y\left( t \right)],$ the MSD is defined as $MSD(\tau)\equiv\frac{1}{t_{max}-\tau}\sum_{t=1}^{t_{max}-\tau} \left( r\left( t+\tau\right)-r\left( t \right) \right)^{2}$, where we have defined the instantaneous modulus as $r\left( t \right)=\sqrt{\left( x\left( t \right) \right)^{2}+(y{\left( t \right))}^{2}}$ and where $\tau$ denotes the time scale. Here, diffusion was studied up to a maximum time scale equal to 1/4th of the data size. An important property of random walks is their power law scaling, $\text{MSD}\left( \tau\right)\text{\textasciitilde}\tau^{\beta}$, where $\beta$ characterizes the behavior of the diffusion process. For uncorrelated Brownian motion, the exponent $\beta$ is equal to 1, when $1<\beta<2$ holds the process is super-diffusive and when $0<\beta<1$ is sub-diffusive. These two processes, super and sub-diffusive, encompass anomalous diffusion, and typically occur in complex systems in the presence of persistence.

Detrended Fluctuation Analysis (DFA)

Detrended Fluctuation Analysis (DFA), a technique introduced by Peng and colleagues, is employed to identify power-law autocorrelations in time series (16). It has found extensive use in the quantification of physiological signals (17). For a given trajectory time series *u*(*t*), the first step involves calculating the signal profile. This is achieved by determining the cumulative sum of the series, represented as $z\left( t \right)=\sum_{k=1}^{t} (u\left( k \right)-<u>)$, where brackets indicate the average of *u*(*k)*. The time series $z\left( t \right)$is then divided into boxes of equal length *n*, and the local trend *z*_n_(*t*) in each box is subtracted. The fluctuation of this detrended signal is calculated by $D\left( n \right)=\sqrt{\frac{1}{t_{max}}\sum_{t=1}^{t_{max}} \left[ z\left( t \right)-z_{n}\left( t \right) \right]^{2}}$. This calculation is performed for all box sizes, resulting in a correlation between fluctuations, denoted as $D$ and box sizes $n$. The presence of power-law autocorrelations is indicated by a linear relationship on a log-log graph, i.e., $D\left( n \right)\sim n^{\gamma}$. In particular, the process exhibits persistence when 0.5< γ <1.

Approximate Entropy

Approximate Entropy (ApEn) is a measure first proposed by Pincus in 1991 (18), which quantifies the regularity and predictability of a time series. A regular signal containing repetitive patterns has a low ApEn, while a complex and hardly predictable series has a high ApEn. The statistic is calculated as follows:

Take a time series of *N* equally spaced values: $U=u(1),u(2),\ldots,u(N)$. The size of the time series can be relatively small (*N*>100) (19). Fix a positive number *r*, and an integer number *m*. The number *r* specifies a filtering level, and a common choice for this parameter is *r* = 0.2·*SD*, where *SD* is the standard deviation of the signal. The optimal value of *m*, the statistic that represents the length of compared runs of data, was estimated by the method proposed by L. Cao (20), and in our case it resulted in *m* = 2. Form a sequence of vectors $x\left( 1 \right), x\left( 2 \right),\ldots,x\left( N-m+1 \right)$ in $\mathbb{R}^{m}$, real m-dimensional space, defined by $x\left( i \right)=[u\left( i \right),\ldots,u(i+m-1)]$. For each $i=1,\ldots,N-m+1$, the sequence $x\left( 1 \right), x\left( 2 \right),\ldots,x\left( N-m+1 \right)$is used to construct

$$C_{i}^{m}\left( r \right)=\frac{number of x\left( j \right) such that d\left[ x\left( i \right), x\left( j \right) \right]\leq r}{\left( N-m+1 \right)}$$

where *d* [*x*(*i*), *x*(*j*)] is defined as $\max_{0\leq k\leq m-1} \{\left| u\left( i+k \right)-u(j+k) \right|\}$.

Thus, *d* represents the distance between the scalar exponents of the vectors *x*(*i*)and *x*(*j*).

Then, the variable $\Phi^{m}\left( r \right)=(N-m+1)^{-1}\sum_{i=1}^{N-m+1} \ln\left( C_{i}^{m}\left( r \right) \right)$ is established, where ln is the natural logarithm. Finally, Approximate Entropy is defined by $ApEn\left( U,r,m \right)=\Phi^{m}\left( r \right)-\Phi^{m-1}\left( r \right)$ for fixed *m* and *r*.

Clustering numerical experiments

The clustering analyses were implemented using our custom code in Python 3.9.13 and Scikit Learn 1.0.2. Combining all experiments across the three cell types (*M. leningradensis*, *A. Proteus*, and *A. borokensis*) and the four experimental conditions (no stimuli, galvanotaxis, chemotaxis, and simultaneous galvanotactic and chemotactic stimuli), unsupervised clustering was performed on all experiments of cellular movement, each defined by a vector of different movement metrics (MM). In the first clustering analysis (Fig. 7), experiments were characterized by the 3D vector of MMs: Intensity of the response (mm), Directionality Ratio, and Average Speed (mm/s). In the second clustering analysis (Fig. 8, experiments were identified using the 5D vector of MMs: RMSF Alpha, RMSF correlation time (move-steps), DFA Gamma, MSD Beta, and Approximate Entropy. Before performing the clustering analysis, and to ensure a comparable visualization to the first analysis, we first applied Principal Component Analyses (PCA) and subsequently applied the clustering strategy to the first three components (PC1, PC2, and PC3), which explained the 92.54% of the total variance in the data. For the second analysis, the clustering results did not change when using RMSF correlation time measured in minutes instead of move steps, as both metrics are highly correlated. Although the best clustering solution (measured by the Silhouette Coefficient) was obtained in both analyses for the number of two clusters, Figs. 7 and 8 display 3 and 4 clusters with the purpose of exploring potential associations with, respectively, cell type and experimental condition. After normalizing the different values using z-scores across all possible experiments, we applied the *k-means* algorithm to obtain 3 and 4 clusters. Subsequently, each cluster was characterized by the proportion of cell types and experimental condition present in each cluster. The performance of the obtained clustering solution was assessed using the *Silhouette Coefficient*, which estimates all the differences between intra-cluster points minus the distances between inter-cluster points. A higher Silhouette index indicates a model with better defined clusters, while a smaller index suggests more heterogeneity in the data, and no well-separated groups. The implementation was achieved using the silhouette score implemented in Scikit-Learn library in Python. To show robustness of the clustering solution and that our results were not dependent on the clustering strategy employed, we repeated the same analyses as in Figs. 7 and 8 but using *hierarchical agglomerative* clustering and depicting the solutions of 3 and 4 clusters (Figs. S3 and S4).

# Statistical analysis

First, the normality of the distribution of our quantitative data was assessed using the Kolmogorov-Smirnoff test for single samples. Given that normality was rejected, the significance of our quantitative results was estimated through the Kruskal-Wallis test for groups and the Wilcoxon rank-sum test for pairs. Since these two are non-parametric tests, results are represented as median/IQ instead of mean ± SD. In addition to the p-values the Z statistics have been reported.

**Supplementary Figures**


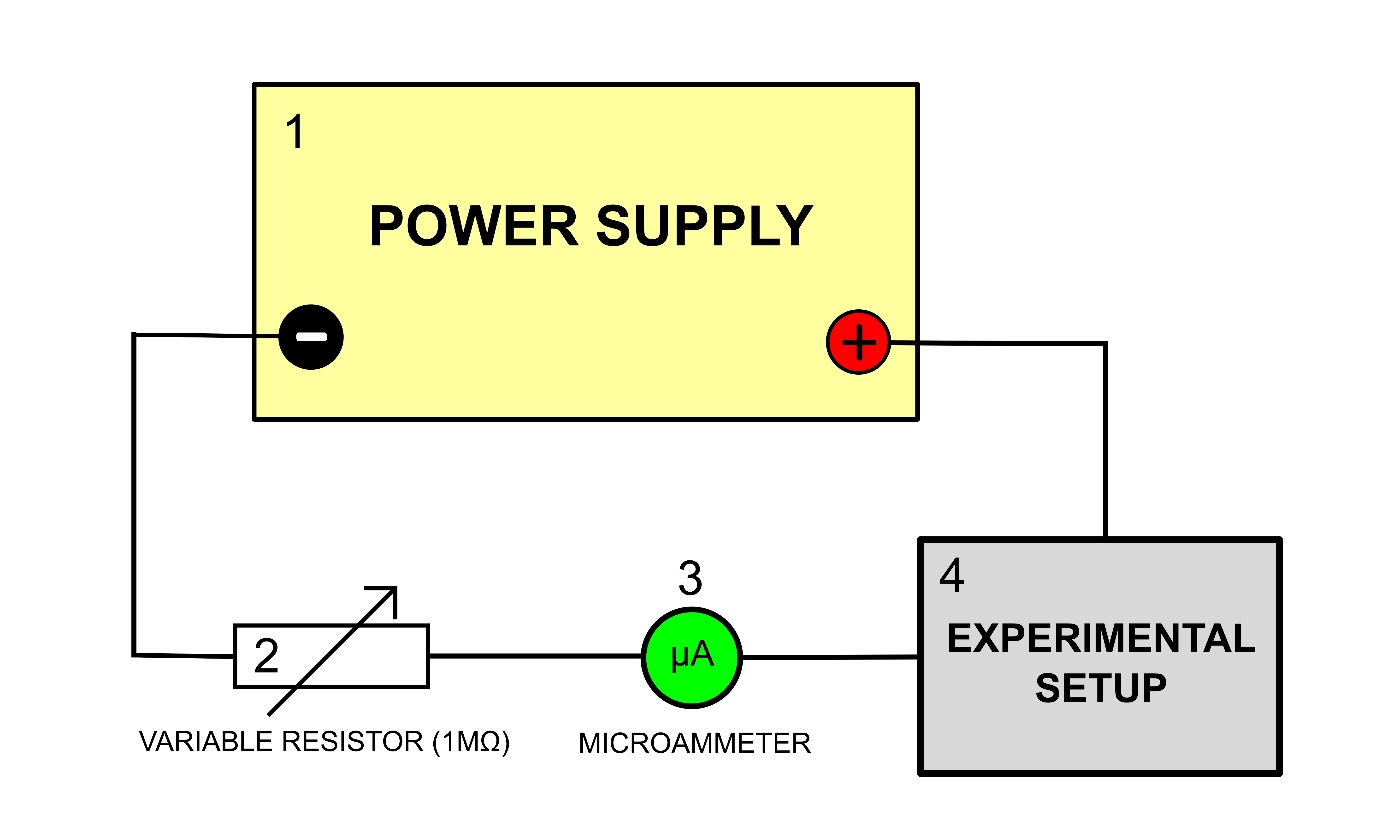


**Figure S1. Auxiliary electric circuitry.**

This circuit allows the electric current that circulates through the experimental system to be monitored and adjusted as necessary. 1: BIO-RAD model 1000/500 power supply unit; 2: 1 MΩ variable resistor to adjust the intensity of the electric current; 3: Microammeter to monitor current intensity flowing through the system; 4: Experimental set-up (see Figures S1 and S2 for more detail).


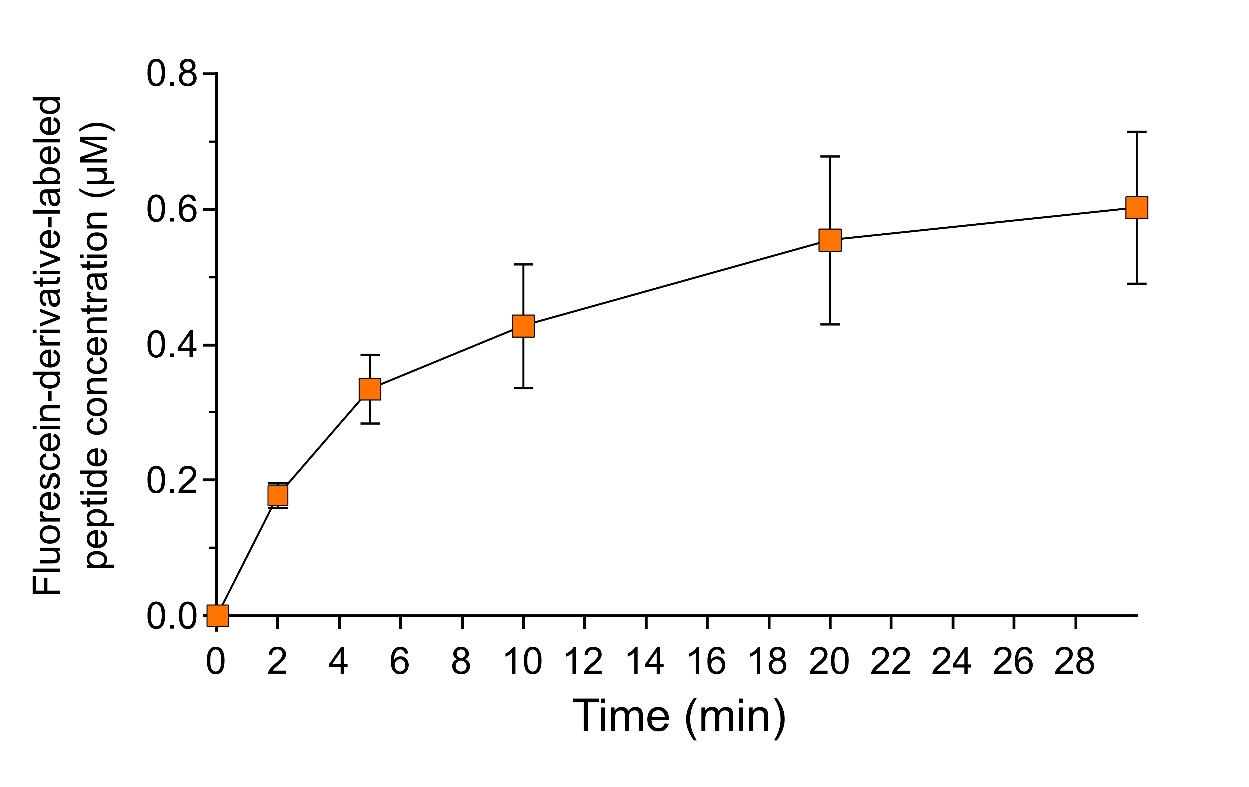


**Figure S2. Peptide gradient concentration measurement.**

Average fluorescein-tagged peptide concentration as a function of time, measured in the center of the experimental glass chamber at 0, 2, 5, 10, 20 and 30 min. Each data point represents the average (± SD) of six measurements (duplicate sampling in three separate experimental replicates) taken at the location where the amoebae were placed. Peptide concentration rises to ~ 0.2 μM two minutes after the laminar flow is established, and further to 0.6 μM towards the end of the experiment (30 min since laminar flow is established).


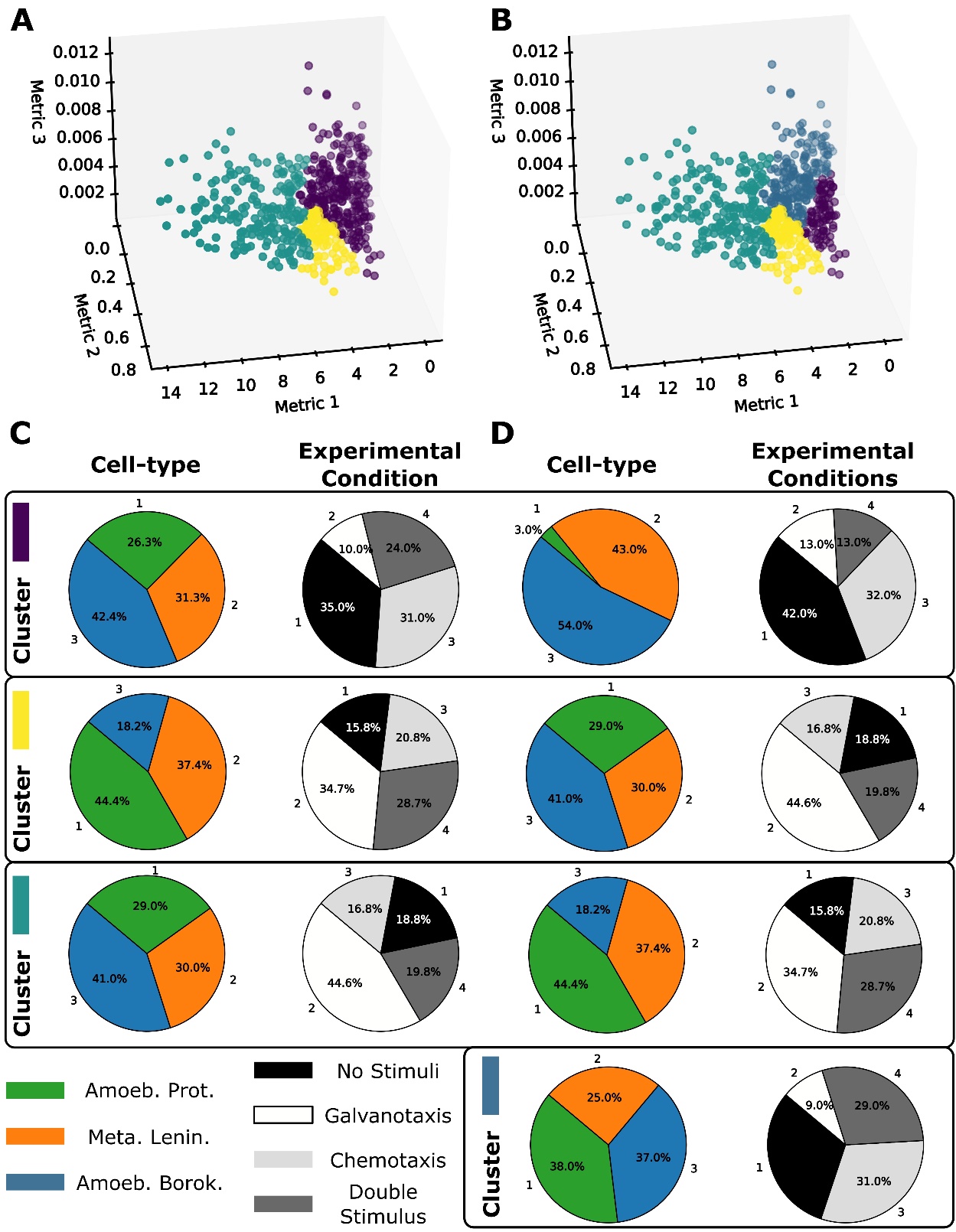


**Figure S3:** Similar to Fig. 7, but employing a different clustering strategy, namely hierarchical agglomerative clustering. The clustering characterization regarding the distinction between cell-types or experimental conditions remains unchanged when varying the clustering strategy, indicating the robustness of the findings.


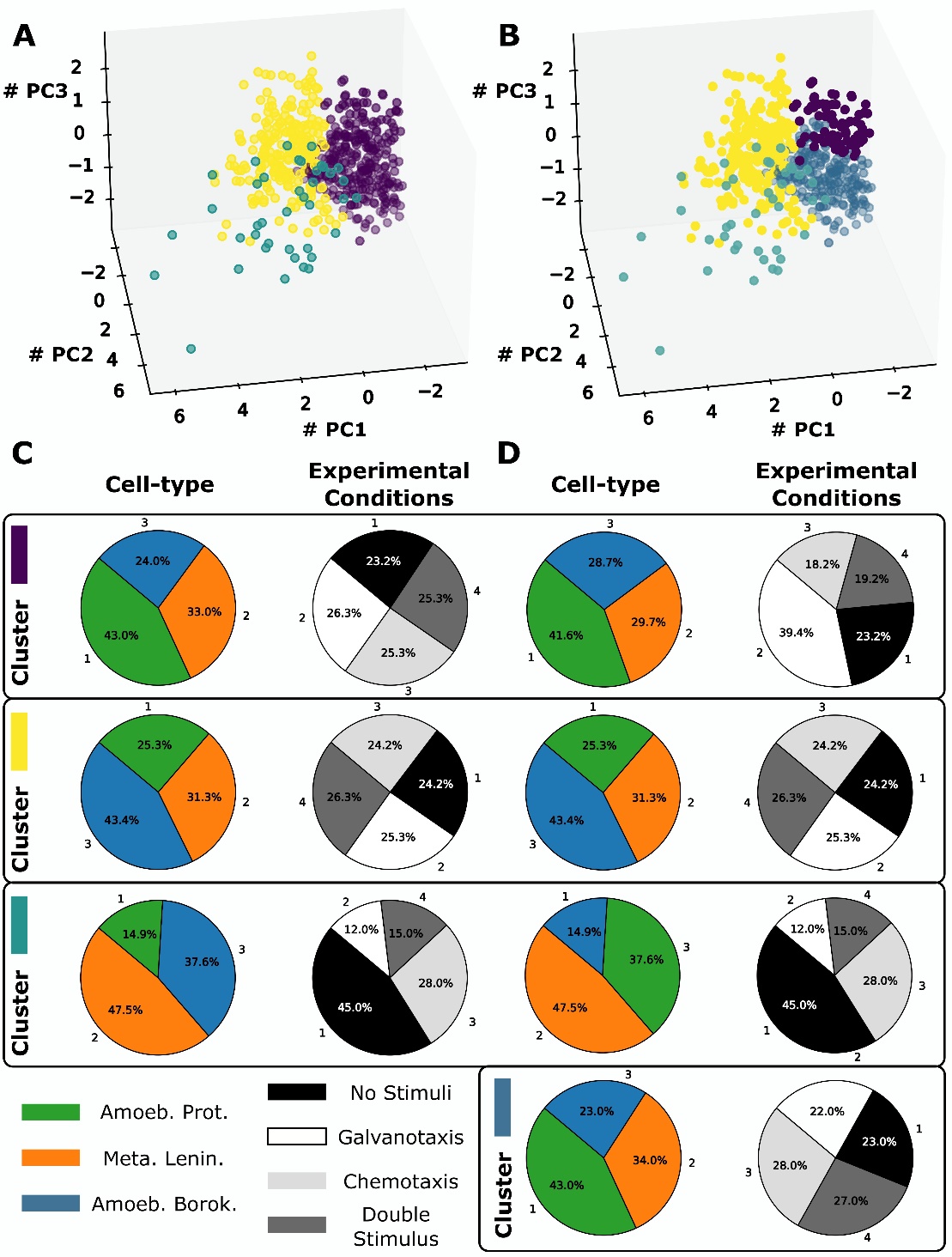


**Figure S4:** Similar to Fig. 8, but employing a different clustering strategy, namely hierarchical agglomerative clustering.

**Supplementary Tables**

**Table S1**

| Cell number | Amoeba proteus | | | | Metamoeba leningradensis | | | | Amoeba borokensis | | | |
| --- | --- | --- | --- | --- | --- | --- | --- | --- | --- | --- | --- | --- |
|  | Sc1 | Sc2 | Sc3 | Sc4 | Sc1 | Sc2 | Sc3 | Sc4 | Sc1 | Sc2 | Sc3 | Sc4 |
| 1 | 0.791 | 0.718 | 0.783 | 0.682 | 0.808 | 0.653 | 0.797 | 0.755 | 0.684 | 0.645 | 0.645 | 0.670 |
| 2 | 0.748 | 0.774 | 0.693 | 0.733 | 0.853 | 0.788 | 0.731 | 0.812 | 0.648 | 0.757 | 0.636 | 0.691 |
| 3 | 0.748 | 0.677 | 0.846 | 0.666 | 0.708 | 0.679 | 0.647 | 0.796 | 0.658 | 0.681 | 0.747 | 0.746 |
| 4 | 0.709 | 0.562 | 0.753 | 0.723 | 0.737 | 0.666 | 0.763 | 0.778 | 0.631 | 0.641 | 0.679 | 0.781 |
| 5 | 0.837 | 0.747 | 0.707 | 0.706 | 0.736 | 0.620 | 0.755 | 0.846 | 0.758 | 0.629 | 0.686 | 0.694 |
| 6 | 0.740 | 0.668 | 0.607 | 0.800 | 0.844 | 0.684 | 0.727 | 0.717 | 0.663 | 0.769 | 0.622 | 0.750 |
| 7 | 0.672 | 0.621 | 0.650 | 0.750 | 0.695 | 0.759 | 0.714 | 0.730 | 0.739 | 0.765 | 0.710 | 0.684 |
| 8 | 0.811 | 0.720 | 0.707 | 0.835 | 0.817 | 0.721 | 0.814 | 0.715 | 0.639 | 0.759 | 0.758 | 0.782 |
| 9 | 0.719 | 0.707 | 0.708 | 0.787 | 0.756 | 0.642 | 0.828 | 0.766 | 0.724 | 0.737 | 0.722 | 0.731 |
| 10 | 0.812 | 0.666 | 0.715 | 0.653 | 0.686 | 0.711 | 0.787 | 0.761 | 0.782 | 0.734 | 0.743 | 0.733 |
| 11 | 0.703 | 0.693 | 0.568 | 0.804 | 0.721 | 0.633 | 0.685 | 0.771 | 0.689 | 0.731 | 0.732 | 0.784 |
| 12 | 0.611 | 0.714 | 0.699 | 0.710 | 0.749 | 0.676 | 0.783 | 0.741 | 0.642 | 0.691 | 0.705 | 0.670 |
| 13 | 0.715 | 0.691 | 0.773 | 0.756 | 0.748 | 0.691 | 0.653 | 0.604 | 0.750 | 0.606 | 0.713 | 0.775 |
| 14 | 0.698 | 0.686 | 0.790 | 0.782 | 0.674 | 0.771 | 0.757 | 0.729 | 0.682 | 0.698 | 0.689 | 0.758 |
| 15 | 0.599 | 0.772 | 0.757 | 0.682 | 0.784 | 0.807 | 0.747 | 0.643 | 0.714 | 0.585 | 0.632 | 0.723 |
| 16 | 0.736 | 0.796 | 0.638 | 0.700 | 0.707 | 0.739 | 0.803 | 0.738 | 0.738 | 0.709 | 0.688 | 0.740 |
| 17 | 0.652 | 0.653 | 0.782 | 0.799 | 0.604 | 0.557 | 0.710 | 0.801 | 0.677 | 0.762 | 0.743 | 0.680 |
| 18 | 0.635 | 0.727 | 0.677 | 0.771 | 0.681 | 0.734 | 0.733 | 0.605 | 0.689 | 0.746 | 0.707 | 0.607 |
| 19 | 0.560 | 0.637 | 0.757 | 0.721 | 0.799 | 0.685 | 0.714 | 0.612 | 0.721 | 0.734 | 0.684 | 0.644 |
| 20 | 0.757 | 0.693 | 0.719 | 0.738 | 0.763 | 0.663 | 0.732 | 0.715 | 0.606 | 0.683 | 0.687 | 0.746 |
| 21 | 0.593 | 0.700 | 0.806 | 0.664 | 0.796 | 0.699 | 0.787 | 0.723 | 0.628 | 0.590 | 0.774 | 0.680 |
| 22 | 0.753 | 0.785 | 0.748 | 0.764 | 0.822 | 0.640 | 0.768 | 0.690 | 0.628 | 0.685 | 0.732 | 0.796 |
| 23 | 0.785 | 0.690 | 0.668 | 0.714 | 0.753 | 0.708 | 0.660 | 0.695 | 0.648 | 0.707 | 0.624 | 0.707 |
| 24 | 0.641 | 0.609 | 0.648 | 0.731 | 0.781 | 0.790 | 0.674 | 0.696 | 0.680 | 0.710 | 0.684 | 0.655 |
| 25 | 0.776 | 0.713 | 0.775 | 0.785 | 0.748 | 0.648 | 0.760 | 0.756 | 0.661 | 0.618 | 0.721 | 0.716 |
| 26 | 0.669 | 0.691 | 0.761 | 0.664 | 0.739 | 0.783 | 0.773 | 0.729 | 0.622 | 0.797 | 0.719 | 0.696 |
| 27 | 0.615 | 0.690 | 0.679 | 0.771 | 0.764 | 0.666 | 0.656 | 0.780 | 0.663 | 0.641 | 0.735 | 0.764 |
| 28 | 0.778 | 0.722 | 0.754 | 0.656 | 0.824 | 0.685 | 0.754 | 0.716 | 0.674 | 0.714 | 0.599 | 0.617 |
| 29 | 0.749 | 0.799 | 0.722 | 0.759 | 0.708 | 0.732 | 0.749 | 0.692 | 0.657 | 0.646 | 0.728 | 0.688 |
| 30 | 0.693 | 0.754 | 0.707 | 0.804 | 0.682 | 0.838 | 0.726 | 0.789 | 0.615 | 0.705 | 0.743 | 0.671 |
| 31 | 0.595 | 0.734 | 0.732 | 0.726 | 0.746 | 0.713 | 0.665 | 0.666 | 0.704 | 0.593 | 0.704 | 0.685 |
| 32 | 0.599 | 0.580 | 0.752 | 0.774 | 0.767 | 0.682 | 0.597 | 0.731 | 0.796 | 0.632 | 0.721 | 0.777 |
| 33 | 0.568 | 0.642 | 0.723 | 0.703 | 0.720 | 0.782 | 0.578 | 0.669 | 0.676 | 0.580 | 0.830 | 0.674 |
| 34 | 0.744 | 0.740 | 0.718 | 0.746 | 0.679 | 0.633 | 0.721 | 0.737 | 0.685 | 0.712 | 0.718 | 0.746 |
| 35 | 0.745 | 0.710 | 0.687 | 0.769 | 0.764 | 0.732 | 0.627 | 0.730 | 0.666 | 0.610 | 0.681 | 0.688 |
| 36 | 0.683 | 0.770 | 0.731 | 0.749 | 0.806 | 0.725 | 0.657 | 0.710 | 0.743 | 0.577 | 0.710 | 0.664 |
| 37 | 0.737 | 0.768 | 0.729 | 0.726 | 0.807 | 0.792 | 0.645 | 0.692 | 0.772 | 0.694 | 0.768 | 0.666 |
| 38 | 0.740 | 0.784 | 0.781 | 0.676 | 0.714 | 0.764 | 0.843 | 0.721 | 0.694 | 0.815 | 0.771 | 0.729 |
| 39 | 0.707 | 0.732 | 0.729 | 0.844 | 0.793 | 0.695 | 0.761 | 0.646 | 0.746 | 0.646 | 0.807 | 0.736 |
| 40 | 0.732 | 0.636 | 0.708 | 0.721 | 0.809 | 0.742 | 0.739 | 0.681 | 0.715 | 0.700 | 0.730 | 0.694 |
| 41 | 0.716 | 0.774 | 0.728 | 0.749 | 0.756 | 0.794 | 0.783 | 0.703 | 0.765 | 0.786 | 0.781 | 0.783 |
| 42 | 0.778 | 0.745 | 0.789 | 0.700 | 0.699 | 0.696 | 0.759 | 0.662 | 0.595 | 0.601 | 0.728 | 0.702 |
| 43 | 0.657 | 0.660 | 0.627 | 0.695 | 0.743 | 0.715 | 0.802 | 0.600 | 0.639 | 0.604 | 0.782 | 0.697 |
| 44 | 0.577 | 0.688 | 0.742 | 0.762 | 0.755 | 0.689 | 0.782 | 0.768 | 0.669 | 0.626 | 0.837 | 0.726 |
| 45 | 0.599 | 0.631 | 0.707 | 0.805 | 0.785 | 0.677 | 0.810 | 0.747 | 0.694 | 0.632 | 0.765 | 0.640 |
| 46 | 0.583 | 0.677 | 0.705 | 0.795 | 0.736 | 0.683 | 0.767 | 0.761 | 0.720 | 0.678 | 0.789 | 0.755 |
| 47 | 0.688 | 0.718 | 0.770 | 0.729 | 0.814 | 0.735 | 0.694 | 0.778 | 0.686 | 0.674 | 0.740 | 0.705 |
| 48 | 0.740 | 0.647 | 0.690 | 0.763 | 0.830 | 0.728 | 0.821 | 0.792 | 0.663 | 0.730 | 0.829 | 0.837 |
| 49 | 0.762 | 0.758 | 0.654 | 0.704 | 0.728 |  | 0.745 | 0.705 | 0.701 | 0.839 | 0.775 | 0.670 |
| 50 | 0.676 |  | 0.741 | 0.725 | 0.845 |  | 0.710 | 0.679 | 0.733 | 0.693 | 0.806 | 0.774 |
| 51 |  |  | 0.745 | 0.678 | 0.722 |  | 0.730 | 0.754 | 0.668 |  | 0.766 | 0.730 |
| 52 |  |  |  | 0.646 |  |  | 0.752 | 0.764 | 0.649 |  | 0.751 | 0.641 |
| 53 |  |  |  | 0.703 |  |  | 0.707 | 0.692 |  |  | 0.786 | 0.723 |
| 54 |  |  |  | 0.609 |  |  | 0.770 | 0.757 |  |  | 0.835 | 0.727 |
| 55 |  |  |  | 0.666 |  |  | 0.683 | 0.848 |  |  | 0.801 | 0.795 |
| 56 |  |  |  | 0.674 |  |  | 0.734 | 0.756 |  |  |  | 0.702 |
| 57 |  |  |  | 0.748 |  |  | 0.685 | 0.828 |  |  |  | 0.752 |
| 58 |  |  |  | 0.794 |  |  | 0.664 | 0.772 |  |  |  | 0.733 |
| 59 |  |  |  | 0.747 |  |  | 0.737 | 0.772 |  |  |  | 0.695 |
| 60 |  |  |  | 0.744 |  |  | 0.630 | 0.654 |  |  |  | 0.728 |
| 61 |  |  |  | 0.810 |  |  |  | 0.722 |  |  |  | 0.684 |
| 62 |  |  |  | 0.799 |  |  |  | 0.692 |  |  |  | 0.687 |
| 63 |  |  |  | 0.671 |  |  |  | 0.746 |  |  |  | 0.678 |
| 64 |  |  |  | 0.867 |  |  |  | 0.806 |  |  |  | 0.768 |
| 65 |  |  |  | 0.783 |  |  |  | 0.775 |  |  |  | 0.758 |
| 66 |  |  |  | 0.664 |  |  |  | 0.747 |  |  |  | 0.732 |
| 67 |  |  |  | 0.713 |  |  |  | 0.691 |  |  |  | 0.810 |
| 68 |  |  |  | 0.713 |  |  |  | 0.689 |  |  |  | 0.737 |
| 69 |  |  |  | 0.760 |  |  |  | 0.690 |  |  |  | 0.707 |
| 70 |  |  |  | 0.647 |  |  |  | 0.812 |  |  |  | 0.776 |
| 71 |  |  |  | 0.735 |  |  |  | 0.827 |  |  |  | 0.794 |
| 72 |  |  |  | 0.823 |  |  |  | 0.766 |  |  |  | 0.700 |
| 73 |  |  |  | 0.663 |  |  |  | 0.802 |  |  |  | 0.751 |
| 74 |  |  |  | 0.733 |  |  |  |  |  |  |  | 0.794 |
| 75 |  |  |  | 0.821 |  |  |  |  |  |  |  | 0.775 |
| 76 |  |  |  | 0.838 |  |  |  |  |  |  |  | 0.840 |
| 77 |  |  |  | 0.808 |  |  |  |  |  |  |  | 0.701 |
| 78 |  |  |  | 0.755 |  |  |  |  |  |  |  | 0.736 |
| 79 |  |  |  | 0.794 |  |  |  |  |  |  |  |  |
| 80 |  |  |  | 0.775 |  |  |  |  |  |  |  |  |
| 81 |  |  |  | 0.727 |  |  |  |  |  |  |  |  |
| 82 |  |  |  | 0.746 |  |  |  |  |  |  |  |  |
| 83 |  |  |  | 0.793 |  |  |  |  |  |  |  |  |

Table S1. Results of “rmsf” analysis (scaling exponent α) of the 700 experimental cell trajectories.

**Table S2**

| Cell number | Amoeba proteus | | | | Metamoeba leningradensis | | | | Amoeba borokensis | | | |
| --- | --- | --- | --- | --- | --- | --- | --- | --- | --- | --- | --- | --- |
|  | Sc1 | Sc2 | Sc3 | Sc4 | Sc1 | Sc2 | Sc3 | Sc4 | Sc1 | Sc2 | Sc3 | Sc4 |
| 1 | 0.433 | 0.477 | 0.440 | 0.423 | 0.473 | 0.453 | 0.477 | 0.553 | 0.484 | 0.493 | 0.436 | 0.459 |
| 2 | 0.463 | 0.517 | 0.492 | 0.539 | 0.474 | 0.448 | 0.533 | 0.407 | 0.548 | 0.449 | 0.415 | 0.449 |
| 3 | 0.446 | 0.538 | 0.469 | 0.500 | 0.584 | 0.493 | 0.434 | 0.449 | 0.485 | 0.466 | 0.513 | 0.448 |
| 4 | 0.502 | 0.523 | 0.428 | 0.502 | 0.459 | 0.478 | 0.490 | 0.466 | 0.391 | 0.462 | 0.429 | 0.413 |
| 5 | 0.540 | 0.452 | 0.516 | 0.422 | 0.448 | 0.529 | 0.435 | 0.467 | 0.467 | 0.463 | 0.515 | 0.467 |
| 6 | 0.460 | 0.544 | 0.457 | 0.474 | 0.423 | 0.414 | 0.410 | 0.494 | 0.407 | 0.508 | 0.509 | 0.488 |
| 7 | 0.414 | 0.495 | 0.502 | 0.595 | 0.432 | 0.450 | 0.494 | 0.487 | 0.444 | 0.589 | 0.512 | 0.457 |
| 8 | 0.533 | 0.473 | 0.400 | 0.459 | 0.468 | 0.452 | 0.509 | 0.400 | 0.539 | 0.475 | 0.451 | 0.481 |
| 9 | 0.583 | 0.460 | 0.389 | 0.455 | 0.393 | 0.381 | 0.470 | 0.583 | 0.528 | 0.590 | 0.644 | 0.456 |
| 10 | 0.459 | 0.435 | 0.453 | 0.479 | 0.538 | 0.511 | 0.411 | 0.467 | 0.446 | 0.515 | 0.456 | 0.472 |
| 11 | 0.471 | 0.397 | 0.490 | 0.400 | 0.431 | 0.418 | 0.460 | 0.534 | 0.431 | 0.479 | 0.399 | 0.504 |
| 12 | 0.386 | 0.487 | 0.479 | 0.417 | 0.448 | 0.424 | 0.485 | 0.433 | 0.531 | 0.473 | 0.498 | 0.470 |
| 13 | 0.516 | 0.475 | 0.408 | 0.489 | 0.479 | 0.365 | 0.470 | 0.451 | 0.481 | 0.568 | 0.556 | 0.461 |
| 14 | 0.432 | 0.509 | 0.566 | 0.545 | 0.492 | 0.495 | 0.425 | 0.422 | 0.398 | 0.532 | 0.369 | 0.471 |
| 15 | 0.573 | 0.546 | 0.390 | 0.393 | 0.486 | 0.501 | 0.429 | 0.490 | 0.480 | 0.449 | 0.445 | 0.470 |
| 16 | 0.525 | 0.487 | 0.464 | 0.502 | 0.472 | 0.456 | 0.505 | 0.480 | 0.643 | 0.460 | 0.468 | 0.484 |
| 17 | 0.423 | 0.535 | 0.545 | 0.401 | 0.492 | 0.444 | 0.488 | 0.481 | 0.516 | 0.547 | 0.447 | 0.507 |
| 18 | 0.477 | 0.504 | 0.420 | 0.408 | 0.361 | 0.437 | 0.527 | 0.373 | 0.565 | 0.455 | 0.487 | 0.403 |
| 19 | 0.452 | 0.444 | 0.380 | 0.438 | 0.476 | 0.526 | 0.383 | 0.401 | 0.384 | 0.432 | 0.424 | 0.418 |
| 20 | 0.472 | 0.525 | 0.436 | 0.520 | 0.421 | 0.499 | 0.399 | 0.418 | 0.442 | 0.460 | 0.454 | 0.521 |
| 21 | 0.575 | 0.476 | 0.436 | 0.425 | 0.428 | 0.595 | 0.368 | 0.393 | 0.559 | 0.536 | 0.455 | 0.479 |
| 22 | 0.397 | 0.591 | 0.475 | 0.530 | 0.502 | 0.492 | 0.538 | 0.391 | 0.601 | 0.477 | 0.561 | 0.438 |
| 23 | 0.488 | 0.467 | 0.504 | 0.461 | 0.476 | 0.397 | 0.530 | 0.585 | 0.446 | 0.569 | 0.475 | 0.495 |
| 24 | 0.511 | 0.541 | 0.430 | 0.381 | 0.480 | 0.575 | 0.587 | 0.476 | 0.484 | 0.469 | 0.386 | 0.442 |
| 25 | 0.454 | 0.393 | 0.465 | 0.422 | 0.454 | 0.415 | 0.434 | 0.369 | 0.435 | 0.476 | 0.480 | 0.436 |
| 26 | 0.440 | 0.494 | 0.527 | 0.555 | 0.464 | 0.418 | 0.392 | 0.405 | 0.449 | 0.368 | 0.523 | 0.545 |
| 27 | 0.471 | 0.397 | 0.453 | 0.502 | 0.487 | 0.433 | 0.432 | 0.412 | 0.473 | 0.515 | 0.593 | 0.482 |
| 28 | 0.505 | 0.441 | 0.436 | 0.433 | 0.448 | 0.468 | 0.504 | 0.428 | 0.422 | 0.490 | 0.453 | 0.437 |
| 29 | 0.502 | 0.486 | 0.405 | 0.498 | 0.481 | 0.555 | 0.432 | 0.462 | 0.470 | 0.569 | 0.476 | 0.483 |
| 30 | 0.397 | 0.477 | 0.525 | 0.449 | 0.516 | 0.522 | 0.498 | 0.520 | 0.447 | 0.477 | 0.400 | 0.473 |
| 31 | 0.500 | 0.433 | 0.454 | 0.435 | 0.384 | 0.421 | 0.502 | 0.455 | 0.464 | 0.467 | 0.477 | 0.491 |
| 32 | 0.480 | 0.400 | 0.477 | 0.408 | 0.392 | 0.536 | 0.568 | 0.482 | 0.458 | 0.421 | 0.459 | 0.438 |
| 33 | 0.498 | 0.482 | 0.492 | 0.587 | 0.476 | 0.495 | 0.621 | 0.476 | 0.503 | 0.438 | 0.493 | 0.489 |
| 34 | 0.478 | 0.421 | 0.454 | 0.519 | 0.450 | 0.497 | 0.543 | 0.419 | 0.596 | 0.475 | 0.430 | 0.434 |
| 35 | 0.426 | 0.562 | 0.416 | 0.443 | 0.517 | 0.405 | 0.578 | 0.543 | 0.391 | 0.423 | 0.510 | 0.482 |
| 36 | 0.432 | 0.490 | 0.475 | 0.374 | 0.494 | 0.378 | 0.510 | 0.439 | 0.482 | 0.420 | 0.435 | 0.445 |
| 37 | 0.477 | 0.444 | 0.415 | 0.444 | 0.384 | 0.442 | 0.573 | 0.462 | 0.379 | 0.477 | 0.462 | 0.395 |
| 38 | 0.487 | 0.449 | 0.437 | 0.596 | 0.483 | 0.477 | 0.412 | 0.531 | 0.476 | 0.467 | 0.480 | 0.413 |
| 39 | 0.455 | 0.469 | 0.473 | 0.477 | 0.404 | 0.435 | 0.431 | 0.427 | 0.476 | 0.448 | 0.409 | 0.459 |
| 40 | 0.516 | 0.557 | 0.514 | 0.483 | 0.490 | 0.461 | 0.388 | 0.495 | 0.503 | 0.451 | 0.535 | 0.550 |
| 41 | 0.567 | 0.422 | 0.455 | 0.432 | 0.403 | 0.510 | 0.400 | 0.470 | 0.504 | 0.451 | 0.440 | 0.481 |
| 42 | 0.488 | 0.442 | 0.510 | 0.477 | 0.436 | 0.504 | 0.492 | 0.534 | 0.444 | 0.431 | 0.409 | 0.422 |
| 43 | 0.444 | 0.436 | 0.462 | 0.476 | 0.419 | 0.484 | 0.448 | 0.483 | 0.463 | 0.485 | 0.427 | 0.416 |
| 44 | 0.448 | 0.449 | 0.503 | 0.410 | 0.568 | 0.454 | 0.472 | 0.414 | 0.551 | 0.491 | 0.524 | 0.476 |
| 45 | 0.477 | 0.502 | 0.488 | 0.508 | 0.393 | 0.444 | 0.466 | 0.501 | 0.487 | 0.504 | 0.415 | 0.429 |
| 46 | 0.529 | 0.461 | 0.459 | 0.422 | 0.466 | 0.550 | 0.451 | 0.486 | 0.451 | 0.424 | 0.484 | 0.501 |
| 47 | 0.354 | 0.426 | 0.527 | 0.425 | 0.458 | 0.429 | 0.611 | 0.441 | 0.423 | 0.473 | 0.559 | 0.518 |
| 48 | 0.431 | 0.480 | 0.442 | 0.523 | 0.406 | 0.460 | 0.441 | 0.365 | 0.466 | 0.548 | 0.562 | 0.509 |
| 49 | 0.497 | 0.498 | 0.535 | 0.472 | 0.441 |  | 0.411 | 0.404 | 0.421 | 0.395 | 0.454 | 0.504 |
| 50 | 0.406 |  | 0.558 | 0.448 | 0.469 |  | 0.407 | 0.425 | 0.432 | 0.536 | 0.446 | 0.556 |
| 51 |  |  | 0.494 | 0.479 | 0.508 |  | 0.479 | 0.520 | 0.497 |  | 0.428 | 0.521 |
| 52 |  |  |  | 0.500 |  |  | 0.617 | 0.522 | 0.480 |  | 0.447 | 0.364 |
| 53 |  |  |  | 0.434 |  |  | 0.563 | 0.469 |  |  | 0.552 | 0.417 |
| 54 |  |  |  | 0.470 |  |  | 0.457 | 0.393 |  |  | 0.368 | 0.503 |
| 55 |  |  |  | 0.451 |  |  | 0.483 | 0.414 |  |  | 0.390 | 0.523 |
| 56 |  |  |  | 0.409 |  |  | 0.453 | 0.465 |  |  |  | 0.490 |
| 57 |  |  |  | 0.527 |  |  | 0.481 | 0.443 |  |  |  | 0.449 |
| 58 |  |  |  | 0.568 |  |  | 0.520 | 0.481 |  |  |  | 0.411 |
| 59 |  |  |  | 0.427 |  |  | 0.404 | 0.408 |  |  |  | 0.483 |
| 60 |  |  |  | 0.476 |  |  | 0.520 | 0.443 |  |  |  | 0.427 |
| 61 |  |  |  | 0.466 |  |  |  | 0.429 |  |  |  | 0.380 |
| 62 |  |  |  | 0.493 |  |  |  | 0.419 |  |  |  | 0.520 |
| 63 |  |  |  | 0.558 |  |  |  | 0.376 |  |  |  | 0.432 |
| 64 |  |  |  | 0.565 |  |  |  | 0.351 |  |  |  | 0.470 |
| 65 |  |  |  | 0.571 |  |  |  | 0.526 |  |  |  | 0.490 |
| 66 |  |  |  | 0.408 |  |  |  | 0.426 |  |  |  | 0.512 |
| 67 |  |  |  | 0.451 |  |  |  | 0.507 |  |  |  | 0.490 |
| 68 |  |  |  | 0.525 |  |  |  | 0.456 |  |  |  | 0.499 |
| 69 |  |  |  | 0.528 |  |  |  | 0.404 |  |  |  | 0.552 |
| 70 |  |  |  | 0.446 |  |  |  | 0.430 |  |  |  | 0.431 |
| 71 |  |  |  | 0.435 |  |  |  | 0.576 |  |  |  | 0.379 |
| 72 |  |  |  | 0.411 |  |  |  | 0.447 |  |  |  | 0.402 |
| 73 |  |  |  | 0.411 |  |  |  | 0.473 |  |  |  | 0.450 |
| 74 |  |  |  | 0.520 |  |  |  |  |  |  |  | 0.515 |
| 75 |  |  |  | 0.446 |  |  |  |  |  |  |  | 0.438 |
| 76 |  |  |  | 0.534 |  |  |  |  |  |  |  | 0.456 |
| 77 |  |  |  | 0.447 |  |  |  |  |  |  |  | 0.516 |
| 78 |  |  |  | 0.549 |  |  |  |  |  |  |  | 0.446 |
| 79 |  |  |  | 0.388 |  |  |  |  |  |  |  |  |
| 80 |  |  |  | 0.457 |  |  |  |  |  |  |  |  |
| 81 |  |  |  | 0.436 |  |  |  |  |  |  |  |  |
| 82 |  |  |  | 0.535 |  |  |  |  |  |  |  |  |
| 83 |  |  |  | 0.613 |  |  |  |  |  |  |  |  |

Table S2. Results of “rmsf” analysis (scaling exponent α) of the 700 shuffled cell trajectories.

**Table S3**

| Cell number | Amoeba proteus | | | | Metamoeba leningradensis | | | | Amoeba borokensis | | | |
| --- | --- | --- | --- | --- | --- | --- | --- | --- | --- | --- | --- | --- |
|  | Sc1 | Sc2 | Sc3 | Sc4 | Sc1 | Sc2 | Sc3 | Sc4 | Sc1 | Sc2 | Sc3 | Sc4 |
| 1 | 2.083 | 14.583 | 11.458 | 10.417 | 13.542 | 15.625 | 15.625 | 4.167 | 14.583 | 10.417 | 14.583 | 5.208 |
| 2 | 13.542 | 15.625 | 6.250 | 5.208 | 12.500 | 7.292 | 7.292 | 10.417 | 12.500 | 8.333 | 8.333 | 5.208 |
| 3 | 14.583 | 11.458 | 14.583 | 4.167 | 5.208 | 7.292 | 16.667 | 14.583 | 9.375 | 11.458 | 10.417 | 4.167 |
| 4 | 8.333 | 7.292 | 8.333 | 3.125 | 7.292 | 12.500 | 5.208 | 9.375 | 6.250 | 3.125 | 13.542 | 2.083 |
| 5 | 9.375 | 15.625 | 5.208 | 14.583 | 6.250 | 3.125 | 13.542 | 13.542 | 14.583 | 4.167 | 2.083 | 11.458 |
| 6 | 10.417 | 15.625 | 11.458 | 5.208 | 11.458 | 4.167 | 8.333 | 10.417 | 4.167 | 14.583 | 4.167 | 3.125 |
| 7 | 7.292 | 16.667 | 15.625 | 4.167 | 4.167 | 15.625 | 6.250 | 13.542 | 13.542 | 3.125 | 10.417 | 11.458 |
| 8 | 12.500 | 15.625 | 9.375 | 13.542 | 13.542 | 6.250 | 6.250 | 6.250 | 10.417 | 4.167 | 7.292 | 3.125 |
| 9 | 5.208 | 8.333 | 3.125 | 7.292 | 4.167 | 5.208 | 6.250 | 8.333 | 9.375 | 10.417 | 8.333 | 4.167 |
| 10 | 5.208 | 16.667 | 8.333 | 14.583 | 8.333 | 7.292 | 12.500 | 8.333 | 15.625 | 13.542 | 6.250 | 6.250 |
| 11 | 3.125 | 13.542 | 8.333 | 14.583 | 11.458 | 14.583 | 6.250 | 6.250 | 7.292 | 15.625 | 7.292 | 7.292 |
| 12 | 13.542 | 13.542 | 10.417 | 12.500 | 5.208 | 7.292 | 14.583 | 6.250 | 14.583 | 6.250 | 15.625 | 3.125 |
| 13 | 5.208 | 5.208 | 11.458 | 12.500 | 4.167 | 7.292 | 11.458 | 7.292 | 13.542 | 6.250 | 6.250 | 4.167 |
| 14 | 5.208 | 12.500 | 8.333 | 14.583 | 14.583 | 7.292 | 3.125 | 6.250 | 15.625 | 10.417 | 4.167 | 4.167 |
| 15 | 13.542 | 10.417 | 6.250 | 10.417 | 14.583 | 11.458 | 13.542 | 3.125 | 2.083 | 8.333 | 13.542 | 4.167 |
| 16 | 2.083 | 10.417 | 12.500 | 7.292 | 8.333 | 10.417 | 2.083 | 5.208 | 12.500 | 3.125 | 6.250 | 9.375 |
| 17 | 14.583 | 7.292 | 9.375 | 12.500 | 10.417 | 15.625 | 4.167 | 5.208 | 3.125 | 7.292 | 8.333 | 7.292 |
| 18 | 3.125 | 7.292 | 13.542 | 3.125 | 12.500 | 5.208 | 3.125 | 10.417 | 2.083 | 4.167 | 12.500 | 11.458 |
| 19 | 9.375 | 15.625 | 12.500 | 11.458 | 11.458 | 13.542 | 4.167 | 14.583 | 6.250 | 9.375 | 11.458 | 14.583 |
| 20 | 13.542 | 14.583 | 9.375 | 3.125 | 5.208 | 13.542 | 5.208 | 4.167 | 8.333 | 12.500 | 7.292 | 12.500 |
| 21 | 12.500 | 2.083 | 11.458 | 11.458 | 7.292 | 11.458 | 15.625 | 6.250 | 6.250 | 2.083 | 7.292 | 14.583 |
| 22 | 15.625 | 11.458 | 7.292 | 10.417 | 10.417 | 13.542 | 3.125 | 14.583 | 6.250 | 12.500 | 6.250 | 3.125 |
| 23 | 13.542 | 2.083 | 5.208 | 10.417 | 12.500 | 7.292 | 3.125 | 13.542 | 3.125 | 4.167 | 12.500 | 9.375 |
| 24 | 15.625 | 2.083 | 4.167 | 8.333 | 10.417 | 15.625 | 13.542 | 6.250 | 4.167 | 5.208 | 5.208 | 5.208 |
| 25 | 10.417 | 9.375 | 10.417 | 13.542 | 2.083 | 14.583 | 11.458 | 7.292 | 5.208 | 13.542 | 8.333 | 10.417 |
| 26 | 6.250 | 5.208 | 10.417 | 16.667 | 8.333 | 9.375 | 5.208 | 6.250 | 14.583 | 14.583 | 5.208 | 8.333 |
| 27 | 14.583 | 14.583 | 9.375 | 4.167 | 13.542 | 8.333 | 13.542 | 14.583 | 9.375 | 2.083 | 15.625 | 15.625 |
| 28 | 14.583 | 1.042 | 13.542 | 11.458 | 13.542 | 9.375 | 8.333 | 13.542 | 2.083 | 11.458 | 10.417 | 9.375 |
| 29 | 14.583 | 11.458 | 14.583 | 12.500 | 15.625 | 10.417 | 5.208 | 6.250 | 7.292 | 7.292 | 6.250 | 8.333 |
| 30 | 10.417 | 12.500 | 5.208 | 13.542 | 16.667 | 14.583 | 6.250 | 10.417 | 8.333 | 8.333 | 7.292 | 5.208 |
| 31 | 7.292 | 13.542 | 7.292 | 13.542 | 9.375 | 10.417 | 12.500 | 12.500 | 4.167 | 12.500 | 8.333 | 7.292 |
| 32 | 3.125 | 15.625 | 12.500 | 13.542 | 12.500 | 8.333 | 14.583 | 12.500 | 15.625 | 4.167 | 4.167 | 3.125 |
| 33 | 14.583 | 11.458 | 12.500 | 10.417 | 15.625 | 7.292 | 9.375 | 10.417 | 2.083 | 5.208 | 10.417 | 11.458 |
| 34 | 13.542 | 15.625 | 6.250 | 12.500 | 15.625 | 7.292 | 15.625 | 12.500 | 6.250 | 14.583 | 8.333 | 9.375 |
| 35 | 9.375 | 6.250 | 9.375 | 10.417 | 8.333 | 2.083 | 4.167 | 5.208 | 11.458 | 16.667 | 6.250 | 13.542 |
| 36 | 8.333 | 13.542 | 8.333 | 9.375 | 15.625 | 11.458 | 12.500 | 3.125 | 14.583 | 11.458 | 12.500 | 12.500 |
| 37 | 9.375 | 8.333 | 11.458 | 13.542 | 9.375 | 15.625 | 4.167 | 6.250 | 5.208 | 9.375 | 7.292 | 5.208 |
| 38 | 9.375 | 15.625 | 10.417 | 10.417 | 8.333 | 11.458 | 4.167 | 5.208 | 14.583 | 14.583 | 13.542 | 10.417 |
| 39 | 9.375 | 13.542 | 11.458 | 10.417 | 9.375 | 14.583 | 12.500 | 10.417 | 6.250 | 13.542 | 11.458 | 10.417 |
| 40 | 7.292 | 11.458 | 7.292 | 10.417 | 10.417 | 6.250 | 13.542 | 10.417 | 3.125 | 3.125 | 7.292 | 5.208 |
| 41 | 15.625 | 15.625 | 9.375 | 9.375 | 6.250 | 13.542 | 7.292 | 6.250 | 11.458 | 8.333 | 4.167 | 10.417 |
| 42 | 6.250 | 2.083 | 8.333 | 4.167 | 3.125 | 13.542 | 11.458 | 4.167 | 7.292 | 8.333 | 6.250 | 8.333 |
| 43 | 15.625 | 4.167 | 11.458 | 2.083 | 5.208 | 6.250 | 9.375 | 5.208 | 6.250 | 7.292 | 12.500 | 3.125 |
| 44 | 15.625 | 5.208 | 7.292 | 6.250 | 8.333 | 6.250 | 13.542 | 3.125 | 9.375 | 7.292 | 15.625 | 13.542 |
| 45 | 10.417 | 6.250 | 15.625 | 6.250 | 9.375 | 4.167 | 14.583 | 12.500 | 5.208 | 7.292 | 7.292 | 8.333 |
| 46 | 2.083 | 15.625 | 11.458 | 9.375 | 10.417 | 11.458 | 10.417 | 14.583 | 2.083 | 3.125 | 10.417 | 4.167 |
| 47 | 7.292 | 13.542 | 5.208 | 10.417 | 7.292 | 12.500 | 7.292 | 14.583 | 15.625 | 4.167 | 5.208 | 10.417 |
| 48 | 10.417 | 15.625 | 12.500 | 12.500 | 14.583 | 9.375 | 15.625 | 13.542 | 13.542 | 7.292 | 11.458 | 3.125 |
| 49 | 14.583 | 15.625 | 14.583 | 7.292 | 6.250 |  | 15.625 | 15.625 | 14.583 | 12.500 | 15.625 | 3.125 |
| 50 | 10.417 |  | 12.500 | 10.417 | 15.625 |  | 8.333 | 13.542 | 11.458 | 6.250 | 8.333 | 3.125 |
| 51 |  |  | 4.167 | 3.125 | 15.625 |  | 6.250 | 15.625 | 7.292 |  | 11.458 | 12.500 |
| 52 |  |  |  | 9.375 |  |  | 14.583 | 11.458 | 10.417 |  | 12.500 | 6.250 |
| 53 |  |  |  | 12.500 |  |  | 10.417 | 13.542 |  |  | 3.125 | 8.333 |
| 54 |  |  |  | 15.625 |  |  | 7.292 | 6.250 |  |  | 14.583 | 8.333 |
| 55 |  |  |  | 11.458 |  |  | 6.250 | 15.625 |  |  | 11.458 | 9.375 |
| 56 |  |  |  | 5.208 |  |  | 8.333 | 15.625 |  |  |  | 3.125 |
| 57 |  |  |  | 11.458 |  |  | 7.292 | 14.583 |  |  |  | 12.500 |
| 58 |  |  |  | 13.542 |  |  | 7.292 | 15.625 |  |  |  | 2.083 |
| 59 |  |  |  | 10.417 |  |  | 3.125 | 15.625 |  |  |  | 7.292 |
| 60 |  |  |  | 10.417 |  |  | 15.625 | 10.417 |  |  |  | 5.208 |
| 61 |  |  |  | 10.417 |  |  |  | 14.583 |  |  |  | 7.292 |
| 62 |  |  |  | 11.458 |  |  |  | 11.458 |  |  |  | 7.292 |
| 63 |  |  |  | 3.125 |  |  |  | 13.542 |  |  |  | 3.125 |
| 64 |  |  |  | 14.583 |  |  |  | 12.500 |  |  |  | 3.125 |
| 65 |  |  |  | 11.458 |  |  |  | 7.292 |  |  |  | 9.375 |
| 66 |  |  |  | 11.458 |  |  |  | 6.250 |  |  |  | 10.417 |
| 67 |  |  |  | 4.167 |  |  |  | 6.250 |  |  |  | 11.458 |
| 68 |  |  |  | 14.583 |  |  |  | 12.500 |  |  |  | 7.292 |
| 69 |  |  |  | 9.375 |  |  |  | 6.250 |  |  |  | 12.500 |
| 70 |  |  |  | 15.625 |  |  |  | 13.542 |  |  |  | 11.458 |
| 71 |  |  |  | 8.333 |  |  |  | 5.208 |  |  |  | 5.208 |
| 72 |  |  |  | 11.458 |  |  |  | 4.167 |  |  |  | 14.583 |
| 73 |  |  |  | 13.542 |  |  |  | 5.208 |  |  |  | 4.167 |
| 74 |  |  |  | 15.625 |  |  |  |  |  |  |  | 7.292 |
| 75 |  |  |  | 14.583 |  |  |  |  |  |  |  | 4.167 |
| 76 |  |  |  | 15.625 |  |  |  |  |  |  |  | 14.583 |
| 77 |  |  |  | 15.625 |  |  |  |  |  |  |  | 4.167 |
| 78 |  |  |  | 8.333 |  |  |  |  |  |  |  | 12.500 |
| 79 |  |  |  | 6.250 |  |  |  |  |  |  |  |  |
| 80 |  |  |  | 4.167 |  |  |  |  |  |  |  |  |
| 81 |  |  |  | 13.542 |  |  |  |  |  |  |  |  |
| 82 |  |  |  | 11.458 |  |  |  |  |  |  |  |  |
| 83 |  |  |  | 14.583 |  |  |  |  |  |  |  |  |

Table S3. Long-range correlation duration values for the 700 experimental cell trajectories.

**Table S4**

| Cell number | Amoeba proteus | | | | Metamoeba leningradensis | | | | Amoeba borokensis | | | |
| --- | --- | --- | --- | --- | --- | --- | --- | --- | --- | --- | --- | --- |
|  | Sc1 | Sc2 | Sc3 | Sc4 | Sc1 | Sc2 | Sc3 | Sc4 | Sc1 | Sc2 | Sc3 | Sc4 |
| 1 | 1.655 | 1.962 | 1.693 | 1.850 | 1.926 | 2.007 | 1.895 | 1.833 | 1.833 | 1.671 | 1.790 | 1.792 |
| 2 | 1.871 | 1.903 | 1.809 | 1.666 | 1.893 | 1.963 | 1.789 | 1.943 | 1.778 | 1.839 | 1.738 | 1.905 |
| 3 | 1.761 | 1.956 | 1.928 | 1.692 | 1.993 | 1.691 | 1.332 | 1.940 | 1.836 | 1.666 | 1.854 | 1.774 |
| 4 | 1.894 | 1.964 | 1.967 | 1.815 | 1.610 | 1.424 | 1.525 | 1.991 | 1.804 | 1.786 | 1.844 | 1.564 |
| 5 | 1.817 | 1.912 | 1.974 | 1.986 | 1.867 | 1.919 | 1.851 | 1.954 | 1.877 | 1.589 | 1.623 | 1.843 |
| 6 | 1.901 | 1.948 | 1.930 | 1.870 | 1.886 | 1.509 | 1.866 | 1.930 | 1.852 | 1.790 | 1.655 | 1.732 |
| 7 | 1.949 | 1.939 | 1.613 | 1.709 | 1.705 | 1.923 | 1.823 | 1.967 | 1.882 | 1.743 | 1.862 | 1.597 |
| 8 | 1.907 | 1.940 | 1.976 | 1.942 | 1.986 | 1.957 | 1.992 | 1.981 | 1.880 | 1.805 | 1.904 | 1.802 |
| 9 | 1.766 | 1.800 | 1.869 | 1.756 | 1.943 | 1.901 | 2.006 | 1.967 | 1.841 | 1.683 | 1.770 | 1.775 |
| 10 | 1.494 | 1.964 | 1.915 | 1.945 | 1.753 | 1.918 | 1.932 | 1.830 | 1.833 | 1.926 | 1.711 | 1.870 |
| 11 | 1.782 | 1.874 | 1.900 | 1.938 | 1.865 | 1.735 | 1.809 | 1.954 | 1.838 | 1.920 | 1.871 | 1.760 |
| 12 | 1.864 | 1.670 | 1.949 | 1.865 | 1.816 | 1.918 | 1.910 | 1.900 | 1.594 | 1.769 | 1.726 | 1.886 |
| 13 | 1.759 | 1.919 | 1.897 | 1.982 | 1.839 | 1.976 | 1.827 | 1.920 | 1.846 | 1.929 | 1.811 | 1.828 |
| 14 | 1.709 | 1.961 | 1.840 | 1.964 | 1.796 | 1.877 | 1.863 | 1.879 | 1.668 | 1.946 | 1.869 | 1.695 |
| 15 | 1.920 | 1.825 | 1.966 | 1.970 | 1.954 | 1.687 | 1.915 | 1.929 | 1.671 | 1.705 | 1.871 | 1.847 |
| 16 | 1.741 | 1.921 | 1.881 | 1.820 | 1.797 | 1.989 | 1.945 | 1.959 | 1.942 | 1.883 | 1.844 | 1.449 |
| 17 | 1.833 | 2.003 | 1.796 | 1.977 | 1.786 | 1.664 | 1.694 | 1.943 | 1.737 | 1.853 | 1.680 | 1.818 |
| 18 | 1.651 | 1.860 | 1.874 | 1.881 | 1.707 | 1.918 | 1.623 | 1.811 | 1.729 | 1.821 | 1.803 | 1.901 |
| 19 | 1.664 | 1.957 | 1.926 | 1.862 | 1.883 | 1.969 | 1.692 | 1.964 | 1.919 | 1.772 | 1.874 | 1.869 |
| 20 | 1.909 | 1.986 | 1.896 | 1.846 | 1.905 | 1.929 | 1.623 | 1.953 | 1.930 | 1.915 | 1.834 | 1.901 |
| 21 | 1.774 | 1.925 | 1.856 | 1.917 | 1.126 | 1.982 | 1.693 | 1.950 | 1.684 | 1.939 | 1.691 | 1.881 |
| 22 | 1.869 | 1.922 | 1.844 | 1.802 | 1.896 | 1.786 | 1.743 | 1.892 | 1.629 | 1.945 | 1.924 | 1.774 |
| 23 | 1.953 | 1.918 | 1.880 | 1.902 | 1.699 | 1.904 | 1.571 | 1.896 | 1.913 | 1.967 | 1.864 | 1.730 |
| 24 | 1.944 | 1.973 | 1.849 | 1.822 | 1.736 | 1.980 | 1.847 | 1.923 | 1.435 | 1.941 | 1.927 | 1.795 |
| 25 | 1.866 | 1.878 | 1.903 | 1.918 | 1.963 | 1.915 | 1.965 | 1.971 | 1.682 | 1.935 | 1.772 | 1.810 |
| 26 | 1.608 | 1.938 | 1.782 | 1.852 | 1.820 | 1.772 | 1.876 | 1.931 | 1.919 | 1.736 | 1.932 | 1.786 |
| 27 | 1.966 | 1.811 | 1.891 | 1.884 | 1.713 | 1.896 | 1.784 | 1.934 | 1.725 | 1.936 | 1.873 | 1.835 |
| 28 | 1.899 | 1.965 | 1.965 | 1.760 | 1.838 | 1.974 | 1.898 | 1.859 | 1.489 | 1.634 | 1.397 | 1.528 |
| 29 | 1.909 | 1.960 | 1.970 | 1.958 | 1.309 | 1.955 | 1.606 | 1.997 | 1.625 | 1.896 | 1.894 | 1.823 |
| 30 | 1.973 | 1.929 | 1.691 | 1.951 | 1.580 | 1.990 | 1.704 | 1.858 | 1.836 | 1.856 | 1.580 | 1.908 |
| 31 | 1.968 | 1.426 | 1.706 | 1.813 | 1.785 | 1.827 | 1.933 | 1.799 | 1.928 | 1.812 | 1.795 | 1.954 |
| 32 | 1.959 | 1.693 | 1.983 | 1.845 | 1.841 | 1.914 | 1.490 | 1.981 | 1.894 | 1.857 | 1.976 | 1.911 |
| 33 | 1.858 | 2.000 | 2.010 | 1.368 | 1.360 | 1.947 | 1.451 | 1.948 | 1.945 | 1.908 | 1.947 | 1.705 |
| 34 | 1.887 | 1.973 | 1.938 | 1.875 | 1.648 | 1.858 | 1.425 | 1.971 | 1.826 | 1.837 | 1.981 | 1.936 |
| 35 | 1.897 | 1.778 | 1.911 | 1.946 | 1.424 | 1.901 | 1.345 | 1.915 | 1.893 | 1.944 | 1.607 | 1.957 |
| 36 | 1.946 | 1.821 | 1.987 | 1.841 | 1.950 | 1.845 | 1.780 | 1.961 | 1.928 | 1.924 | 1.902 | 1.863 |
| 37 | 1.906 | 1.829 | 1.978 | 1.899 | 1.642 | 1.870 | 1.194 | 1.944 | 1.892 | 1.870 | 1.641 | 1.696 |
| 38 | 1.990 | 1.954 | 1.913 | 1.893 | 1.707 | 1.892 | 1.767 | 1.987 | 2.006 | 1.918 | 1.967 | 1.608 |
| 39 | 1.807 | 1.961 | 1.943 | 1.939 | 1.627 | 1.922 | 1.804 | 1.979 | 1.947 | 1.891 | 1.848 | 1.720 |
| 40 | 1.948 | 1.969 | 1.826 | 1.902 | 1.474 | 1.918 | 1.764 | 1.903 | 1.805 | 1.834 | 1.739 | 1.773 |
| 41 | 1.894 | 1.925 | 1.936 | 1.831 | 1.560 | 1.986 | 1.799 | 1.926 | 1.834 | 1.915 | 1.928 | 1.712 |
| 42 | 1.925 | 1.877 | 1.916 | 1.756 | 1.618 | 1.895 | 1.785 | 1.989 | 1.904 | 1.928 | 1.781 | 1.783 |
| 43 | 1.944 | 1.992 | 1.923 | 1.794 | 1.770 | 1.858 | 1.711 | 1.962 | 1.708 | 1.974 | 1.763 | 1.629 |
| 44 | 1.516 | 1.899 | 1.785 | 1.889 | 1.853 | 1.820 | 1.856 | 1.905 | 1.810 | 1.882 | 1.933 | 1.803 |
| 45 | 1.979 | 1.968 | 1.245 | 1.821 | 1.926 | 1.885 | 1.921 | 1.827 | 1.706 | 1.957 | 1.977 | 1.577 |
| 46 | 1.806 | 1.949 | 1.795 | 1.979 | 1.882 | 1.718 | 1.841 | 1.944 | 1.092 | 1.923 | 1.993 | 1.630 |
| 47 | 1.887 | 1.971 | 1.784 | 1.965 | 1.695 | 1.792 | 1.787 | 1.940 | 1.943 | 1.946 | 1.999 | 1.820 |
| 48 | 1.891 | 1.983 | 1.890 | 1.962 | 1.821 | 1.962 | 1.956 | 1.923 | 1.823 | 1.912 | 1.964 | 1.688 |
| 49 | 1.950 | 1.973 | 1.833 | 1.617 | 1.951 |  | 1.872 | 1.810 | 1.895 | 1.901 | 1.960 | 1.825 |
| 50 | 1.876 |  | 1.860 | 1.939 | 1.936 |  | 1.869 | 1.571 | 1.951 | 1.910 | 1.823 | 1.838 |
| 51 |  |  | 1.792 | 1.829 | 1.810 |  | 1.642 | 1.880 | 1.931 |  | 1.942 | 1.845 |
| 52 |  |  |  | 1.752 |  |  | 1.868 | 1.907 | 1.797 |  | 1.837 | 1.979 |
| 53 |  |  |  | 1.857 |  |  | 1.963 | 1.856 |  |  | 1.763 | 1.965 |
| 54 |  |  |  | 1.566 |  |  | 1.936 | 1.916 |  |  | 1.970 | 1.863 |
| 55 |  |  |  | 1.748 |  |  | 1.775 | 1.959 |  |  | 1.861 | 1.950 |
| 56 |  |  |  | 1.687 |  |  | 1.765 | 1.898 |  |  |  | 1.868 |
| 57 |  |  |  | 1.935 |  |  | 1.694 | 1.888 |  |  |  | 1.909 |
| 58 |  |  |  | 1.890 |  |  | 1.645 | 1.957 |  |  |  | 1.842 |
| 59 |  |  |  | 1.934 |  |  | 1.364 | 1.896 |  |  |  | 2.002 |
| 60 |  |  |  | 1.600 |  |  | 1.431 | 1.729 |  |  |  | 1.846 |
| 61 |  |  |  | 1.864 |  |  |  | 1.904 |  |  |  | 1.551 |
| 62 |  |  |  | 1.884 |  |  |  | 1.874 |  |  |  | 1.903 |
| 63 |  |  |  | 1.935 |  |  |  | 1.725 |  |  |  | 1.693 |
| 64 |  |  |  | 1.923 |  |  |  | 1.938 |  |  |  | 1.612 |
| 65 |  |  |  | 1.940 |  |  |  | 1.978 |  |  |  | 1.770 |
| 66 |  |  |  | 1.950 |  |  |  | 1.840 |  |  |  | 1.717 |
| 67 |  |  |  | 1.675 |  |  |  | 1.441 |  |  |  | 1.862 |
| 68 |  |  |  | 1.865 |  |  |  | 1.749 |  |  |  | 1.939 |
| 69 |  |  |  | 1.916 |  |  |  | 1.976 |  |  |  | 1.995 |
| 70 |  |  |  | 1.723 |  |  |  | 1.891 |  |  |  | 2.022 |
| 71 |  |  |  | 1.907 |  |  |  | 1.837 |  |  |  | 1.890 |
| 72 |  |  |  | 1.972 |  |  |  | 1.878 |  |  |  | 1.785 |
| 73 |  |  |  | 1.778 |  |  |  | 1.709 |  |  |  | 1.936 |
| 74 |  |  |  | 1.929 |  |  |  |  |  |  |  | 1.925 |
| 75 |  |  |  | 1.811 |  |  |  |  |  |  |  | 1.870 |
| 76 |  |  |  | 1.916 |  |  |  |  |  |  |  | 1.907 |
| 77 |  |  |  | 1.968 |  |  |  |  |  |  |  | 1.787 |
| 78 |  |  |  | 1.980 |  |  |  |  |  |  |  | 1.902 |
| 79 |  |  |  | 1.958 |  |  |  |  |  |  |  |  |
| 80 |  |  |  | 1.849 |  |  |  |  |  |  |  |  |
| 81 |  |  |  | 1.860 |  |  |  |  |  |  |  |  |
| 82 |  |  |  | 1.870 |  |  |  |  |  |  |  |  |
| 83 |  |  |  | 1.949 |  |  |  |  |  |  |  |  |

Table S4. Results of MSD analysis of the 700 experimental cell trajectories.

**Table S5**

| Cell number | Amoeba proteus | | | | Metamoeba leningradensis | | | | Amoeba borokensis | | | |
| --- | --- | --- | --- | --- | --- | --- | --- | --- | --- | --- | --- | --- |
|  | Sc1 | Sc2 | Sc3 | Sc4 | Sc1 | Sc2 | Sc3 | Sc4 | Sc1 | Sc2 | Sc3 | Sc4 |
| 1 | −0.001 | 0.001 | 0.001 | −0.001 | −0.001 | 0.001 | 0.002 | 0.001 | 0.001 | 0.003 | −0.004 | 0.000 |
| 2 | 0.005 | 0.000 | −0.001 | −0.002 | 0.002 | 0.001 | −0.002 | −0.001 | 0.003 | −0.002 | −0.003 | 0.000 |
| 3 | −0.004 | −0.001 | −0.002 | −0.002 | 0.001 | −0.001 | 0.003 | 0.002 | 0.001 | −0.001 | −0.001 | −0.002 |
| 4 | 0.002 | −0.001 | 0.000 | −0.001 | 0.003 | 0.000 | 0.002 | 0.000 | 0.005 | −0.003 | 0.000 | 0.003 |
| 5 | 0.000 | 0.001 | 0.001 | 0.001 | 0.001 | 0.001 | 0.001 | 0.000 | 0.000 | 0.001 | −0.005 | 0.000 |
| 6 | −0.002 | 0.002 | −0.002 | −0.003 | 0.000 | 0.000 | 0.002 | 0.000 | −0.001 | −0.001 | 0.000 | 0.001 |
| 7 | −0.002 | −0.003 | −0.001 | 0.001 | 0.000 | −0.002 | 0.001 | 0.001 | 0.003 | 0.000 | 0.001 | −0.006 |
| 8 | −0.001 | 0.001 | 0.001 | 0.000 | −0.004 | 0.002 | 0.001 | −0.001 | 0.000 | −0.001 | −0.003 | 0.000 |
| 9 | −0.001 | 0.002 | −0.001 | −0.001 | 0.001 | 0.002 | 0.001 | −0.002 | 0.000 | −0.001 | 0.001 | 0.003 |
| 10 | 0.002 | −0.002 | 0.000 | 0.002 | 0.000 | −0.002 | 0.001 | 0.000 | 0.003 | −0.002 | −0.001 | 0.001 |
| 11 | 0.001 | −0.001 | 0.002 | −0.001 | 0.001 | −0.001 | 0.002 | 0.000 | 0.001 | 0.000 | 0.001 | 0.000 |
| 12 | −0.001 | 0.003 | 0.001 | 0.001 | 0.000 | −0.002 | 0.001 | 0.001 | −0.001 | −0.001 | −0.001 | 0.001 |
| 13 | −0.003 | −0.001 | −0.001 | 0.000 | 0.002 | 0.000 | 0.002 | −0.003 | 0.001 | −0.001 | −0.002 | 0.000 |
| 14 | 0.003 | −0.004 | −0.001 | −0.001 | −0.003 | 0.002 | −0.001 | 0.002 | −0.003 | 0.000 | 0.000 | 0.002 |
| 15 | 0.002 | 0.003 | 0.002 | −0.001 | 0.001 | −0.003 | 0.000 | −0.001 | 0.000 | 0.002 | −0.002 | −0.001 |
| 16 | −0.001 | −0.002 | 0.000 | 0.002 | −0.002 | 0.002 | 0.002 | −0.003 | −0.003 | 0.000 | 0.000 | 0.003 |
| 17 | −0.001 | 0.001 | 0.000 | 0.000 | −0.003 | −0.003 | 0.000 | 0.002 | −0.002 | 0.000 | −0.001 | 0.000 |
| 18 | −0.001 | 0.000 | 0.001 | 0.002 | 0.002 | 0.000 | 0.000 | −0.001 | 0.002 | 0.000 | 0.002 | −0.001 |
| 19 | 0.002 | 0.000 | 0.000 | 0.002 | −0.003 | 0.004 | 0.002 | −0.001 | −0.001 | −0.001 | −0.001 | 0.000 |
| 20 | −0.004 | 0.001 | −0.001 | 0.003 | −0.003 | 0.002 | 0.002 | 0.001 | −0.003 | −0.001 | 0.003 | 0.003 |
| 21 | 0.002 | −0.004 | −0.001 | −0.002 | −0.004 | −0.001 | 0.002 | −0.001 | −0.002 | −0.001 | −0.003 | −0.002 |
| 22 | 0.002 | 0.000 | 0.001 | 0.002 | 0.002 | 0.000 | 0.002 | 0.002 | −0.002 | 0.002 | 0.001 | −0.001 |
| 23 | 0.000 | −0.001 | 0.000 | −0.003 | 0.000 | 0.001 | 0.001 | −0.003 | −0.001 | 0.002 | −0.001 | −0.002 |
| 24 | −0.001 | 0.000 | 0.001 | −0.002 | 0.000 | 0.001 | −0.001 | 0.005 | 0.000 | 0.001 | 0.002 | −0.003 |
| 25 | 0.001 | 0.001 | −0.001 | 0.001 | 0.000 | 0.002 | 0.000 | 0.002 | −0.001 | 0.003 | 0.002 | 0.000 |
| 26 | 0.005 | −0.001 | 0.003 | 0.000 | 0.001 | −0.001 | −0.001 | 0.001 | 0.001 | 0.002 | 0.000 | 0.002 |
| 27 | 0.003 | 0.001 | −0.001 | 0.000 | 0.006 | 0.001 | 0.003 | −0.001 | −0.002 | −0.001 | 0.001 | 0.000 |
| 28 | 0.001 | 0.000 | −0.004 | 0.002 | −0.001 | 0.001 | 0.001 | 0.000 | 0.002 | 0.000 | −0.001 | −0.003 |
| 29 | −0.002 | −0.001 | 0.001 | −0.001 | −0.001 | 0.000 | −0.001 | −0.002 | 0.003 | 0.004 | 0.000 | −0.001 |
| 30 | 0.000 | −0.001 | −0.002 | −0.001 | 0.002 | 0.000 | −0.002 | 0.003 | 0.000 | 0.003 | 0.002 | 0.003 |
| 31 | 0.000 | 0.001 | 0.000 | 0.001 | 0.002 | −0.003 | 0.001 | −0.002 | 0.000 | 0.001 | 0.003 | 0.000 |
| 32 | −0.001 | 0.000 | 0.001 | −0.001 | 0.001 | −0.002 | 0.004 | 0.002 | −0.003 | −0.001 | 0.000 | −0.004 |
| 33 | 0.001 | −0.001 | −0.001 | −0.003 | 0.002 | 0.002 | 0.000 | 0.002 | 0.000 | 0.002 | −0.001 | 0.002 |
| 34 | −0.002 | 0.000 | −0.003 | 0.001 | 0.001 | −0.002 | 0.000 | 0.000 | −0.001 | 0.001 | 0.001 | −0.001 |
| 35 | 0.006 | 0.000 | −0.002 | 0.001 | −0.006 | −0.001 | 0.003 | 0.002 | −0.001 | 0.000 | 0.005 | 0.002 |
| 36 | 0.000 | 0.002 | 0.002 | 0.001 | 0.000 | −0.007 | 0.000 | 0.000 | 0.002 | 0.002 | 0.002 | 0.000 |
| 37 | −0.001 | −0.002 | −0.001 | 0.001 | 0.001 | 0.001 | −0.001 | −0.001 | −0.001 | −0.001 | 0.001 | 0.000 |
| 38 | 0.000 | −0.004 | −0.003 | 0.000 | 0.000 | −0.001 | 0.000 | −0.001 | 0.000 | −0.001 | 0.000 | −0.003 |
| 39 | −0.001 | −0.002 | 0.004 | 0.000 | −0.001 | 0.000 | 0.002 | 0.001 | 0.001 | 0.000 | −0.001 | 0.000 |
| 40 | −0.002 | 0.001 | 0.000 | −0.001 | 0.001 | −0.001 | 0.003 | 0.002 | 0.003 | 0.003 | 0.000 | −0.001 |
| 41 | 0.004 | −0.001 | −0.002 | 0.001 | 0.001 | −0.001 | 0.001 | 0.002 | −0.001 | 0.002 | 0.003 | −0.002 |
| 42 | 0.001 | −0.001 | 0.001 | 0.001 | 0.006 | 0.001 | 0.000 | 0.000 | −0.001 | 0.000 | −0.002 | −0.001 |
| 43 | 0.001 | 0.000 | 0.001 | 0.003 | −0.001 | 0.001 | −0.007 | 0.001 | −0.001 | 0.000 | 0.001 | −0.001 |
| 44 | 0.002 | 0.001 | −0.002 | −0.002 | −0.001 | −0.001 | −0.001 | 0.000 | 0.003 | 0.000 | 0.000 | 0.001 |
| 45 | −0.004 | 0.002 | −0.001 | 0.001 | 0.000 | −0.001 | 0.000 | −0.002 | −0.002 | −0.002 | 0.001 | −0.001 |
| 46 | 0.001 | −0.001 | 0.000 | −0.002 | −0.002 | 0.001 | −0.002 | 0.000 | 0.001 | 0.000 | 0.001 | −0.001 |
| 47 | 0.000 | 0.001 | −0.002 | −0.002 | −0.003 | 0.002 | 0.001 | 0.002 | 0.002 | 0.001 | −0.002 | 0.000 |
| 48 | −0.001 | 0.001 | 0.000 | 0.000 | 0.002 | 0.000 | 0.000 | −0.003 | −0.002 | 0.001 | −0.001 | 0.002 |
| 49 | 0.001 | 0.001 | 0.001 | −0.001 | 0.000 |  | −0.002 | −0.001 | −0.001 | 0.000 | −0.004 | −0.002 |
| 50 | 0.001 |  | −0.001 | 0.000 | 0.002 |  | 0.001 | 0.002 | 0.001 | 0.000 | −0.003 | −0.002 |
| 51 |  |  | 0.000 | −0.001 | 0.001 |  | 0.003 | 0.000 | 0.001 |  | −0.001 | −0.003 |
| 52 |  |  |  | 0.000 |  |  | 0.004 | −0.003 | 0.002 |  | −0.002 | 0.002 |
| 53 |  |  |  | 0.001 |  |  | 0.002 | −0.003 |  |  | −0.001 | −0.002 |
| 54 |  |  |  | 0.000 |  |  | −0.001 | −0.001 |  |  | 0.001 | 0.001 |
| 55 |  |  |  | −0.001 |  |  | 0.001 | −0.001 |  |  | 0.001 | 0.000 |
| 56 |  |  |  | −0.002 |  |  | 0.001 | 0.002 |  |  |  | 0.001 |
| 57 |  |  |  | 0.001 |  |  | −0.002 | 0.001 |  |  |  | 0.002 |
| 58 |  |  |  | 0.001 |  |  | −0.002 | 0.003 |  |  |  | 0.001 |
| 59 |  |  |  | 0.001 |  |  | −0.002 | −0.001 |  |  |  | 0.000 |
| 60 |  |  |  | 0.002 |  |  | −0.002 | 0.001 |  |  |  | 0.000 |
| 61 |  |  |  | 0.000 |  |  |  | −0.002 |  |  |  | 0.003 |
| 62 |  |  |  | 0.000 |  |  |  | −0.001 |  |  |  | −0.001 |
| 63 |  |  |  | 0.001 |  |  |  | 0.001 |  |  |  | 0.002 |
| 64 |  |  |  | 0.000 |  |  |  | 0.000 |  |  |  | −0.003 |
| 65 |  |  |  | −0.001 |  |  |  | 0.000 |  |  |  | 0.001 |
| 66 |  |  |  | −0.002 |  |  |  | −0.003 |  |  |  | −0.003 |
| 67 |  |  |  | −0.001 |  |  |  | −0.001 |  |  |  | 0.001 |
| 68 |  |  |  | 0.002 |  |  |  | −0.001 |  |  |  | 0.002 |
| 69 |  |  |  | 0.000 |  |  |  | 0.000 |  |  |  | −0.001 |
| 70 |  |  |  | −0.002 |  |  |  | −0.001 |  |  |  | 0.000 |
| 71 |  |  |  | −0.002 |  |  |  | 0.002 |  |  |  | 0.001 |
| 72 |  |  |  | 0.000 |  |  |  | 0.000 |  |  |  | −0.003 |
| 73 |  |  |  | −0.002 |  |  |  | −0.001 |  |  |  | 0.001 |
| 74 |  |  |  | 0.001 |  |  |  |  |  |  |  | −0.001 |
| 75 |  |  |  | −0.003 |  |  |  |  |  |  |  | 0.003 |
| 76 |  |  |  | 0.000 |  |  |  |  |  |  |  | 0.003 |
| 77 |  |  |  | 0.000 |  |  |  |  |  |  |  | 0.003 |
| 78 |  |  |  | 0.000 |  |  |  |  |  |  |  | −0.004 |
| 79 |  |  |  | 0.000 |  |  |  |  |  |  |  |  |
| 80 |  |  |  | −0.001 |  |  |  |  |  |  |  |  |
| 81 |  |  |  | −0.002 |  |  |  |  |  |  |  |  |
| 82 |  |  |  | 0.001 |  |  |  |  |  |  |  |  |
| 83 |  |  |  | 0.000 |  |  |  |  |  |  |  |  |

Table S5. Results of MSD analysis of the 700 shuffled cell trajectories.

**Table S6**

| Cell number | Amoeba proteus | | | | Metamoeba leningradensis | | | | Amoeba borokensis | | | |
| --- | --- | --- | --- | --- | --- | --- | --- | --- | --- | --- | --- | --- |
|  | Sc1 | Sc2 | Sc3 | Sc4 | Sc1 | Sc2 | Sc3 | Sc4 | Sc1 | Sc2 | Sc3 | Sc4 |
| 1 | 0.012 | 0.002 | 0.006 | 0.003 | 0.003 | 0.001 | 0.002 | 0.003 | 0.003 | 0.006 | 0.005 | 0.004 |
| 2 | 0.002 | 0.002 | 0.004 | 0.010 | 0.003 | 0.002 | 0.002 | 0.002 | 0.004 | 0.004 | 0.004 | 0.002 |
| 3 | 0.006 | 0.001 | 0.003 | 0.006 | 0.001 | 0.004 | 0.022 | 0.003 | 0.002 | 0.005 | 0.004 | 0.003 |
| 4 | 0.002 | 0.001 | 0.002 | 0.004 | 0.012 | 0.009 | 0.012 | 0.001 | 0.007 | 0.004 | 0.003 | 0.008 |
| 5 | 0.005 | 0.002 | 0.002 | 0.002 | 0.003 | 0.003 | 0.002 | 0.001 | 0.002 | 0.007 | 0.011 | 0.003 |
| 6 | 0.002 | 0.002 | 0.003 | 0.002 | 0.002 | 0.008 | 0.006 | 0.002 | 0.007 | 0.008 | 0.009 | 0.004 |
| 7 | 0.006 | 0.003 | 0.010 | 0.004 | 0.004 | 0.003 | 0.003 | 0.001 | 0.003 | 0.003 | 0.003 | 0.007 |
| 8 | 0.002 | 0.003 | 0.002 | 0.002 | 0.001 | 0.003 | 0.002 | 0.001 | 0.004 | 0.002 | 0.002 | 0.004 |
| 9 | 0.005 | 0.004 | 0.004 | 0.004 | 0.003 | 0.003 | 0.001 | 0.001 | 0.005 | 0.004 | 0.003 | 0.003 |
| 10 | 0.012 | 0.003 | 0.003 | 0.002 | 0.005 | 0.003 | 0.003 | 0.005 | 0.004 | 0.002 | 0.003 | 0.008 |
| 11 | 0.009 | 0.003 | 0.003 | 0.003 | 0.003 | 0.004 | 0.005 | 0.002 | 0.004 | 0.002 | 0.003 | 0.004 |
| 12 | 0.002 | 0.007 | 0.002 | 0.003 | 0.005 | 0.002 | 0.002 | 0.002 | 0.008 | 0.003 | 0.008 | 0.003 |
| 13 | 0.003 | 0.003 | 0.002 | 0.001 | 0.003 | 0.002 | 0.005 | 0.002 | 0.003 | 0.002 | 0.007 | 0.008 |
| 14 | 0.005 | 0.002 | 0.002 | 0.002 | 0.003 | 0.003 | 0.002 | 0.004 | 0.004 | 0.002 | 0.004 | 0.007 |
| 15 | 0.003 | 0.005 | 0.002 | 0.002 | 0.002 | 0.004 | 0.001 | 0.003 | 0.005 | 0.005 | 0.003 | 0.003 |
| 16 | 0.007 | 0.002 | 0.002 | 0.003 | 0.004 | 0.002 | 0.002 | 0.004 | 0.002 | 0.003 | 0.003 | 0.014 |
| 17 | 0.004 | 0.002 | 0.002 | 0.002 | 0.003 | 0.005 | 0.005 | 0.001 | 0.011 | 0.002 | 0.005 | 0.004 |
| 18 | 0.006 | 0.004 | 0.003 | 0.003 | 0.007 | 0.003 | 0.004 | 0.003 | 0.009 | 0.003 | 0.003 | 0.002 |
| 19 | 0.007 | 0.003 | 0.004 | 0.005 | 0.002 | 0.001 | 0.005 | 0.002 | 0.002 | 0.003 | 0.002 | 0.003 |
| 20 | 0.004 | 0.002 | 0.003 | 0.003 | 0.004 | 0.001 | 0.009 | 0.001 | 0.003 | 0.004 | 0.003 | 0.003 |
| 21 | 0.005 | 0.002 | 0.003 | 0.002 | 0.024 | 0.002 | 0.009 | 0.001 | 0.007 | 0.003 | 0.006 | 0.002 |
| 22 | 0.005 | 0.002 | 0.003 | 0.004 | 0.003 | 0.003 | 0.007 | 0.003 | 0.007 | 0.003 | 0.002 | 0.004 |
| 23 | 0.002 | 0.002 | 0.002 | 0.002 | 0.003 | 0.003 | 0.007 | 0.002 | 0.003 | 0.002 | 0.004 | 0.004 |
| 24 | 0.003 | 0.002 | 0.003 | 0.004 | 0.008 | 0.001 | 0.002 | 0.001 | 0.013 | 0.003 | 0.002 | 0.003 |
| 25 | 0.002 | 0.002 | 0.002 | 0.002 | 0.003 | 0.003 | 0.001 | 0.002 | 0.004 | 0.003 | 0.007 | 0.003 |
| 26 | 0.007 | 0.002 | 0.006 | 0.003 | 0.004 | 0.005 | 0.003 | 0.001 | 0.003 | 0.005 | 0.001 | 0.003 |
| 27 | 0.002 | 0.002 | 0.005 | 0.004 | 0.004 | 0.003 | 0.004 | 0.001 | 0.008 | 0.003 | 0.003 | 0.004 |
| 28 | 0.006 | 0.002 | 0.002 | 0.004 | 0.003 | 0.002 | 0.003 | 0.004 | 0.008 | 0.010 | 0.013 | 0.006 |
| 29 | 0.004 | 0.002 | 0.002 | 0.002 | 0.014 | 0.002 | 0.004 | 0.001 | 0.006 | 0.003 | 0.003 | 0.003 |
| 30 | 0.003 | 0.002 | 0.009 | 0.001 | 0.009 | 0.002 | 0.004 | 0.004 | 0.003 | 0.003 | 0.014 | 0.003 |
| 31 | 0.003 | 0.012 | 0.006 | 0.003 | 0.008 | 0.004 | 0.001 | 0.004 | 0.002 | 0.004 | 0.003 | 0.001 |
| 32 | 0.003 | 0.005 | 0.002 | 0.002 | 0.004 | 0.003 | 0.017 | 0.001 | 0.003 | 0.003 | 0.001 | 0.002 |
| 33 | 0.005 | 0.002 | 0.001 | 0.007 | 0.017 | 0.002 | 0.014 | 0.002 | 0.002 | 0.003 | 0.002 | 0.007 |
| 34 | 0.006 | 0.002 | 0.002 | 0.002 | 0.012 | 0.003 | 0.009 | 0.002 | 0.007 | 0.004 | 0.001 | 0.001 |
| 35 | 0.003 | 0.004 | 0.003 | 0.002 | 0.009 | 0.002 | 0.010 | 0.002 | 0.002 | 0.003 | 0.009 | 0.001 |
| 36 | 0.002 | 0.006 | 0.002 | 0.003 | 0.003 | 0.005 | 0.003 | 0.001 | 0.002 | 0.003 | 0.006 | 0.004 |
| 37 | 0.002 | 0.005 | 0.003 | 0.003 | 0.006 | 0.002 | 0.019 | 0.001 | 0.002 | 0.004 | 0.004 | 0.006 |
| 38 | 0.002 | 0.002 | 0.002 | 0.003 | 0.005 | 0.002 | 0.003 | 0.002 | 0.002 | 0.003 | 0.002 | 0.006 |
| 39 | 0.005 | 0.002 | 0.002 | 0.003 | 0.011 | 0.002 | 0.003 | 0.002 | 0.002 | 0.003 | 0.006 | 0.005 |
| 40 | 0.003 | 0.002 | 0.003 | 0.002 | 0.013 | 0.002 | 0.003 | 0.003 | 0.004 | 0.003 | 0.007 | 0.003 |
| 41 | 0.004 | 0.001 | 0.002 | 0.003 | 0.010 | 0.002 | 0.007 | 0.003 | 0.003 | 0.002 | 0.002 | 0.005 |
| 42 | 0.002 | 0.002 | 0.002 | 0.005 | 0.006 | 0.003 | 0.008 | 0.002 | 0.002 | 0.003 | 0.007 | 0.004 |
| 43 | 0.002 | 0.001 | 0.002 | 0.003 | 0.004 | 0.003 | 0.005 | 0.002 | 0.005 | 0.002 | 0.003 | 0.005 |
| 44 | 0.015 | 0.002 | 0.003 | 0.002 | 0.006 | 0.003 | 0.003 | 0.002 | 0.010 | 0.003 | 0.002 | 0.003 |
| 45 | 0.002 | 0.001 | 0.015 | 0.003 | 0.002 | 0.003 | 0.002 | 0.004 | 0.008 | 0.002 | 0.001 | 0.014 |
| 46 | 0.004 | 0.001 | 0.004 | 0.001 | 0.005 | 0.004 | 0.002 | 0.002 | 0.018 | 0.002 | 0.001 | 0.005 |
| 47 | 0.003 | 0.001 | 0.007 | 0.002 | 0.006 | 0.004 | 0.002 | 0.002 | 0.003 | 0.002 | 0.001 | 0.003 |
| 48 | 0.006 | 0.001 | 0.003 | 0.002 | 0.004 | 0.001 | 0.002 | 0.002 | 0.004 | 0.002 | 0.001 | 0.008 |
| 49 | 0.003 | 0.001 | 0.004 | 0.005 | 0.001 |  | 0.003 | 0.004 | 0.003 | 0.003 | 0.002 | 0.003 |
| 50 | 0.005 |  | 0.003 | 0.002 | 0.002 |  | 0.003 | 0.006 | 0.002 | 0.002 | 0.002 | 0.002 |
| 51 |  |  | 0.003 | 0.004 | 0.003 |  | 0.005 | 0.003 | 0.002 |  | 0.001 | 0.003 |
| 52 |  |  |  | 0.004 |  |  | 0.002 | 0.002 | 0.003 |  | 0.006 | 0.001 |
| 53 |  |  |  | 0.003 |  |  | 0.002 | 0.002 |  |  | 0.005 | 0.001 |
| 54 |  |  |  | 0.014 |  |  | 0.002 | 0.002 |  |  | 0.002 | 0.003 |
| 55 |  |  |  | 0.004 |  |  | 0.005 | 0.001 |  |  | 0.004 | 0.002 |
| 56 |  |  |  | 0.006 |  |  | 0.004 | 0.002 |  |  |  | 0.003 |
| 57 |  |  |  | 0.003 |  |  | 0.006 | 0.002 |  |  |  | 0.002 |
| 58 |  |  |  | 0.004 |  |  | 0.006 | 0.001 |  |  |  | 0.005 |
| 59 |  |  |  | 0.003 |  |  | 0.013 | 0.003 |  |  |  | 0.001 |
| 60 |  |  |  | 0.006 |  |  | 0.011 | 0.004 |  |  |  | 0.003 |
| 61 |  |  |  | 0.008 |  |  |  | 0.003 |  |  |  | 0.009 |
| 62 |  |  |  | 0.002 |  |  |  | 0.004 |  |  |  | 0.002 |
| 63 |  |  |  | 0.002 |  |  |  | 0.004 |  |  |  | 0.004 |
| 64 |  |  |  | 0.002 |  |  |  | 0.003 |  |  |  | 0.009 |
| 65 |  |  |  | 0.001 |  |  |  | 0.002 |  |  |  | 0.007 |
| 66 |  |  |  | 0.002 |  |  |  | 0.003 |  |  |  | 0.006 |
| 67 |  |  |  | 0.004 |  |  |  | 0.007 |  |  |  | 0.005 |
| 68 |  |  |  | 0.002 |  |  |  | 0.004 |  |  |  | 0.001 |
| 69 |  |  |  | 0.002 |  |  |  | 0.002 |  |  |  | 0.000 |
| 70 |  |  |  | 0.005 |  |  |  | 0.003 |  |  |  | 0.001 |
| 71 |  |  |  | 0.003 |  |  |  | 0.002 |  |  |  | 0.001 |
| 72 |  |  |  | 0.003 |  |  |  | 0.002 |  |  |  | 0.005 |
| 73 |  |  |  | 0.004 |  |  |  | 0.003 |  |  |  | 0.001 |
| 74 |  |  |  | 0.002 |  |  |  |  |  |  |  | 0.001 |
| 75 |  |  |  | 0.004 |  |  |  |  |  |  |  | 0.002 |
| 76 |  |  |  | 0.001 |  |  |  |  |  |  |  | 0.001 |
| 77 |  |  |  | 0.002 |  |  |  |  |  |  |  | 0.002 |
| 78 |  |  |  | 0.001 |  |  |  |  |  |  |  | 0.001 |
| 79 |  |  |  | 0.002 |  |  |  |  |  |  |  |  |
| 80 |  |  |  | 0.002 |  |  |  |  |  |  |  |  |
| 81 |  |  |  | 0.003 |  |  |  |  |  |  |  |  |
| 82 |  |  |  | 0.003 |  |  |  |  |  |  |  |  |
| 83 |  |  |  | 0.002 |  |  |  |  |  |  |  |  |

Table S6. Results of Approximate Entropy estimation for the 700 experimental cell trajectories.

**Table S7**

| Cell number | Amoeba proteus | | | | Metamoeba leningradensis | | | | Amoeba borokensis | | | |
| --- | --- | --- | --- | --- | --- | --- | --- | --- | --- | --- | --- | --- |
|  | Sc1 | Sc2 | Sc3 | Sc4 | Sc1 | Sc2 | Sc3 | Sc4 | Sc1 | Sc2 | Sc3 | Sc4 |
| 1 | 2.071 | 1.964 | 1.853 | 1.857 | 1.968 | 2.067 | 1.613 | 1.927 | 1.688 | 1.896 | 1.933 | 2.045 |
| 2 | 1.849 | 1.901 | 2.004 | 1.952 | 1.762 | 1.892 | 1.963 | 2.062 | 1.922 | 1.879 | 1.840 | 2.049 |
| 3 | 1.740 | 2.097 | 1.683 | 1.826 | 2.090 | 1.974 | 2.006 | 1.901 | 1.890 | 1.669 | 1.605 | 1.970 |
| 4 | 2.009 | 2.077 | 1.947 | 1.897 | 1.830 | 1.894 | 2.000 | 1.886 | 1.988 | 2.033 | 1.871 | 1.995 |
| 5 | 1.892 | 2.031 | 2.079 | 2.015 | 1.914 | 2.079 | 1.770 | 1.656 | 1.747 | 1.960 | 2.057 | 1.761 |
| 6 | 1.979 | 1.943 | 1.953 | 1.928 | 1.574 | 1.992 | 1.982 | 1.864 | 2.041 | 1.788 | 1.836 | 1.957 |
| 7 | 2.053 | 1.986 | 2.076 | 2.015 | 1.960 | 2.004 | 1.998 | 2.016 | 1.979 | 1.807 | 1.681 | 1.813 |
| 8 | 1.700 | 2.036 | 2.028 | 1.650 | 2.007 | 2.023 | 1.991 | 2.070 | 1.908 | 1.996 | 1.806 | 2.076 |
| 9 | 1.938 | 1.856 | 1.993 | 1.763 | 1.960 | 2.074 | 1.916 | 2.003 | 1.941 | 1.911 | 1.752 | 1.993 |
| 10 | 2.003 | 2.033 | 2.032 | 1.991 | 1.996 | 2.021 | 1.823 | 1.989 | 1.608 | 1.917 | 1.789 | 2.055 |
| 11 | 2.006 | 2.034 | 2.115 | 1.762 | 1.832 | 1.931 | 1.977 | 1.962 | 1.891 | 1.984 | 1.826 | 1.859 |
| 12 | 1.931 | 1.732 | 2.027 | 1.814 | 1.958 | 1.999 | 2.003 | 1.878 | 1.734 | 2.008 | 1.660 | 2.088 |
| 13 | 1.936 | 1.969 | 1.955 | 2.046 | 1.996 | 2.069 | 1.984 | 1.904 | 2.010 | 2.102 | 1.997 | 2.003 |
| 14 | 2.028 | 2.074 | 2.008 | 1.973 | 1.636 | 2.022 | 2.024 | 1.606 | 2.026 | 2.099 | 1.881 | 1.976 |
| 15 | 2.093 | 1.815 | 2.026 | 1.976 | 2.085 | 1.595 | 2.008 | 2.079 | 1.895 | 1.807 | 2.090 | 1.830 |
| 16 | 2.013 | 2.036 | 2.050 | 1.833 | 1.699 | 2.049 | 2.065 | 2.019 | 1.891 | 1.950 | 1.945 | 1.928 |
| 17 | 2.083 | 2.046 | 1.992 | 1.849 | 2.023 | 1.978 | 1.918 | 2.059 | 2.031 | 1.983 | 1.867 | 2.013 |
| 18 | 1.865 | 1.922 | 1.957 | 1.984 | 1.894 | 2.080 | 1.981 | 1.972 | 2.032 | 2.037 | 1.888 | 2.079 |
| 19 | 1.809 | 2.043 | 1.960 | 1.745 | 1.893 | 2.081 | 1.953 | 2.096 | 2.039 | 1.960 | 1.944 | 1.942 |
| 20 | 1.866 | 2.036 | 1.973 | 1.963 | 1.981 | 2.108 | 2.039 | 2.108 | 2.086 | 1.912 | 2.005 | 2.018 |
| 21 | 1.885 | 2.104 | 1.851 | 2.037 | 1.766 | 2.041 | 1.969 | 2.082 | 1.859 | 2.093 | 1.804 | 1.925 |
| 22 | 1.857 | 1.745 | 1.945 | 1.734 | 1.809 | 1.865 | 2.049 | 2.081 | 1.912 | 2.097 | 2.032 | 1.920 |
| 23 | 1.912 | 2.097 | 1.960 | 2.075 | 1.252 | 2.106 | 1.821 | 1.971 | 2.051 | 2.042 | 1.956 | 1.968 |
| 24 | 1.901 | 2.098 | 1.862 | 1.862 | 1.954 | 2.051 | 1.919 | 2.065 | 1.879 | 2.014 | 2.076 | 2.066 |
| 25 | 1.923 | 2.053 | 1.895 | 1.826 | 2.055 | 2.062 | 1.982 | 1.953 | 1.897 | 2.101 | 1.999 | 1.870 |
| 26 | 1.969 | 2.115 | 1.747 | 2.053 | 1.976 | 1.852 | 1.999 | 2.072 | 1.952 | 1.818 | 2.106 | 1.929 |
| 27 | 2.081 | 1.834 | 1.991 | 1.989 | 1.428 | 2.088 | 1.856 | 2.052 | 1.869 | 2.070 | 1.821 | 1.613 |
| 28 | 2.052 | 2.066 | 2.007 | 1.862 | 1.357 | 2.039 | 1.877 | 1.985 | 1.974 | 1.898 | 1.972 | 2.053 |
| 29 | 1.918 | 1.888 | 2.022 | 2.001 | 1.728 | 1.969 | 1.924 | 2.081 | 1.797 | 2.085 | 1.956 | 1.979 |
| 30 | 2.001 | 2.006 | 1.899 | 1.855 | 1.605 | 2.014 | 1.759 | 1.960 | 1.858 | 2.066 | 1.896 | 2.037 |
| 31 | 2.051 | 1.589 | 1.803 | 1.860 | 1.881 | 2.018 | 1.970 | 1.830 | 1.965 | 1.973 | 1.995 | 2.048 |
| 32 | 2.074 | 1.922 | 1.899 | 1.941 | 1.885 | 2.030 | 1.999 | 2.055 | 1.653 | 2.062 | 2.055 | 2.054 |
| 33 | 2.037 | 2.036 | 1.990 | 1.671 | 1.835 | 2.007 | 2.015 | 1.963 | 2.077 | 2.100 | 1.800 | 1.952 |
| 34 | 1.947 | 2.066 | 2.088 | 1.904 | 1.988 | 2.098 | 1.942 | 1.901 | 2.006 | 1.860 | 2.021 | 2.041 |
| 35 | 2.039 | 1.961 | 1.890 | 1.879 | 1.579 | 2.072 | 1.890 | 1.957 | 1.903 | 2.091 | 1.738 | 1.997 |
| 36 | 2.085 | 1.956 | 2.013 | 1.898 | 1.749 | 1.897 | 1.819 | 2.094 | 1.916 | 2.054 | 2.081 | 1.980 |
| 37 | 2.080 | 2.034 | 1.872 | 1.896 | 1.754 | 1.697 | 2.016 | 2.075 | 1.964 | 2.088 | 1.773 | 1.707 |
| 38 | 2.013 | 2.054 | 1.894 | 2.080 | 1.844 | 1.990 | 1.782 | 2.003 | 2.016 | 1.951 | 1.913 | 1.786 |
| 39 | 1.827 | 2.028 | 1.969 | 1.677 | 1.919 | 2.078 | 1.811 | 2.036 | 1.981 | 2.079 | 1.920 | 1.901 |
| 40 | 2.029 | 2.087 | 1.910 | 1.826 | 1.945 | 1.975 | 1.543 | 1.999 | 1.834 | 2.057 | 2.011 | 1.968 |
| 41 | 1.843 | 2.077 | 1.986 | 1.780 | 1.890 | 1.980 | 1.912 | 1.830 | 1.977 | 2.077 | 1.879 | 1.778 |
| 42 | 1.927 | 2.092 | 1.822 | 1.934 | 1.610 | 2.018 | 1.998 | 2.026 | 2.126 | 1.921 | 2.059 | 1.896 |
| 43 | 2.053 | 2.064 | 2.043 | 2.049 | 1.714 | 1.963 | 1.835 | 2.008 | 2.059 | 2.068 | 1.749 | 1.928 |
| 44 | 1.952 | 2.057 | 1.862 | 1.810 | 1.935 | 2.012 | 1.651 | 2.001 | 2.082 | 2.072 | 1.711 | 2.032 |
| 45 | 2.075 | 2.085 | 1.967 | 1.899 | 1.884 | 2.017 | 1.763 | 1.699 | 1.953 | 2.089 | 2.075 | 1.841 |
| 46 | 1.931 | 2.075 | 1.999 | 1.967 | 1.921 | 1.916 | 1.824 | 1.915 | 1.995 | 2.065 | 1.968 | 2.011 |
| 47 | 2.102 | 2.053 | 1.809 | 1.909 | 1.743 | 1.927 | 1.994 | 1.942 | 1.884 | 2.026 | 2.061 | 2.093 |
| 48 | 2.007 | 2.019 | 1.791 | 1.857 | 1.514 | 2.050 | 1.904 | 1.916 | 1.986 | 1.848 | 1.968 | 1.999 |
| 49 | 2.011 | 2.050 | 2.060 | 1.971 | 2.055 |  | 1.820 | 1.934 | 1.967 | 1.880 | 1.950 | 1.986 |
| 50 | 1.831 |  | 1.870 | 1.824 | 1.628 |  | 1.896 | 1.762 | 1.893 | 2.070 | 1.672 | 1.919 |
| 51 |  |  | 1.833 | 1.974 | 1.913 |  | 1.799 | 1.984 | 2.044 |  | 2.045 | 1.821 |
| 52 |  |  |  | 1.873 |  |  | 1.934 | 1.996 | 2.033 |  | 1.809 | 2.045 |
| 53 |  |  |  | 1.689 |  |  | 2.059 | 2.004 |  |  | 1.906 | 2.015 |
| 54 |  |  |  | 1.940 |  |  | 1.805 | 2.059 |  |  | 1.671 | 1.929 |
| 55 |  |  |  | 1.772 |  |  | 1.905 | 1.925 |  |  | 1.963 | 1.831 |
| 56 |  |  |  | 1.963 |  |  | 1.796 | 2.009 |  |  |  | 1.985 |
| 57 |  |  |  | 2.060 |  |  | 1.780 | 1.811 |  |  |  | 1.943 |
| 58 |  |  |  | 1.799 |  |  | 1.630 | 2.038 |  |  |  | 2.016 |
| 59 |  |  |  | 1.992 |  |  | 2.046 | 1.950 |  |  |  | 2.040 |
| 60 |  |  |  | 2.003 |  |  | 1.943 | 1.979 |  |  |  | 1.922 |
| 61 |  |  |  | 1.921 |  |  |  | 1.904 |  |  |  | 1.860 |
| 62 |  |  |  | 1.993 |  |  |  | 1.958 |  |  |  | 2.058 |
| 63 |  |  |  | 2.092 |  |  |  | 1.832 |  |  |  | 1.973 |
| 64 |  |  |  | 1.606 |  |  |  | 1.922 |  |  |  | 1.894 |
| 65 |  |  |  | 1.924 |  |  |  | 1.956 |  |  |  | 2.013 |
| 66 |  |  |  | 2.003 |  |  |  | 1.982 |  |  |  | 1.925 |
| 67 |  |  |  | 1.849 |  |  |  | 2.057 |  |  |  | 1.941 |
| 68 |  |  |  | 1.816 |  |  |  | 1.937 |  |  |  | 1.987 |
| 69 |  |  |  | 1.974 |  |  |  | 1.998 |  |  |  | 2.084 |
| 70 |  |  |  | 1.908 |  |  |  | 1.840 |  |  |  | 2.009 |
| 71 |  |  |  | 1.993 |  |  |  | 1.979 |  |  |  | 2.082 |
| 72 |  |  |  | 1.731 |  |  |  | 2.083 |  |  |  | 1.898 |
| 73 |  |  |  | 1.694 |  |  |  | 1.962 |  |  |  | 2.088 |
| 74 |  |  |  | 1.973 |  |  |  |  |  |  |  | 2.007 |
| 75 |  |  |  | 1.552 |  |  |  |  |  |  |  | 1.972 |
| 76 |  |  |  | 1.668 |  |  |  |  |  |  |  | 1.854 |
| 77 |  |  |  | 1.980 |  |  |  |  |  |  |  | 1.928 |
| 78 |  |  |  | 2.037 |  |  |  |  |  |  |  | 1.994 |
| 79 |  |  |  | 1.993 |  |  |  |  |  |  |  |  |
| 80 |  |  |  | 1.769 |  |  |  |  |  |  |  |  |
| 81 |  |  |  | 1.863 |  |  |  |  |  |  |  |  |
| 82 |  |  |  | 1.822 |  |  |  |  |  |  |  |  |
| 83 |  |  |  | 1.804 |  |  |  |  |  |  |  |  |

Table S7. Results of Approximate Entropy estimation for the 700 shuffled cell trajectories.

**Table S8**

| Cell number | Amoeba proteus | | | | Metamoeba leningradensis | | | | Amoeba borokensis | | | |
| --- | --- | --- | --- | --- | --- | --- | --- | --- | --- | --- | --- | --- |
|  | Sc1 | Sc2 | Sc3 | Sc4 | Sc1 | Sc2 | Sc3 | Sc4 | Sc1 | Sc2 | Sc3 | Sc4 |
| 1 | 1.644 | 1.794 | 1.715 | 1.746 | 1.704 | 1.844 | 1.830 | 1.783 | 1.827 | 1.539 | 1.803 | 1.736 |
| 2 | 1.805 | 1.670 | 1.645 | 1.688 | 1.562 | 1.840 | 1.755 | 1.766 | 1.568 | 1.674 | 1.748 | 1.771 |
| 3 | 1.778 | 1.781 | 1.868 | 1.468 | 1.814 | 1.671 | 1.350 | 1.873 | 1.818 | 1.714 | 1.869 | 1.784 |
| 4 | 1.800 | 1.809 | 1.856 | 1.809 | 1.518 | 1.521 | 1.518 | 1.862 | 1.618 | 1.741 | 1.799 | 1.639 |
| 5 | 1.736 | 1.716 | 1.818 | 1.802 | 1.828 | 1.820 | 1.799 | 1.881 | 1.832 | 1.535 | 1.551 | 1.800 |
| 6 | 1.686 | 1.857 | 1.826 | 1.790 | 1.793 | 1.582 | 1.860 | 1.824 | 1.799 | 1.707 | 1.770 | 1.723 |
| 7 | 1.895 | 1.820 | 1.536 | 1.626 | 1.589 | 1.762 | 1.787 | 1.845 | 1.745 | 1.747 | 1.873 | 1.314 |
| 8 | 1.826 | 1.764 | 1.828 | 1.693 | 1.846 | 1.832 | 1.853 | 1.821 | 1.827 | 1.703 | 1.875 | 1.776 |
| 9 | 1.750 | 1.745 | 1.668 | 1.770 | 1.839 | 1.796 | 1.870 | 1.840 | 1.772 | 1.502 | 1.764 | 1.776 |
| 10 | 1.426 | 1.818 | 1.784 | 1.781 | 1.753 | 1.823 | 1.900 | 1.787 | 1.821 | 1.747 | 1.677 | 1.736 |
| 11 | 1.739 | 1.816 | 1.780 | 1.757 | 1.836 | 1.680 | 1.856 | 1.813 | 1.781 | 1.767 | 1.842 | 1.703 |
| 12 | 1.820 | 1.723 | 1.848 | 1.735 | 1.786 | 1.807 | 1.761 | 1.831 | 1.738 | 1.770 | 1.802 | 1.748 |
| 13 | 1.633 | 1.842 | 1.801 | 1.830 | 1.793 | 1.832 | 1.816 | 1.839 | 1.794 | 1.798 | 1.644 | 1.672 |
| 14 | 1.497 | 1.799 | 1.745 | 1.755 | 1.799 | 1.775 | 1.692 | 1.870 | 1.707 | 1.793 | 1.846 | 1.824 |
| 15 | 1.772 | 1.689 | 1.836 | 1.875 | 1.827 | 1.370 | 1.737 | 1.780 | 1.633 | 1.782 | 1.785 | 1.822 |
| 16 | 1.805 | 1.791 | 1.732 | 1.851 | 1.776 | 1.858 | 1.816 | 1.881 | 1.849 | 1.822 | 1.775 | 1.324 |
| 17 | 1.740 | 1.854 | 1.662 | 1.805 | 1.767 | 1.688 | 1.691 | 1.814 | 1.659 | 1.771 | 1.761 | 1.789 |
| 18 | 1.650 | 1.780 | 1.654 | 1.820 | 1.690 | 1.816 | 1.665 | 1.717 | 1.701 | 1.657 | 1.743 | 1.801 |
| 19 | 1.445 | 1.814 | 1.872 | 1.857 | 1.607 | 1.828 | 1.627 | 1.794 | 1.824 | 1.716 | 1.836 | 1.672 |
| 20 | 1.851 | 1.827 | 1.823 | 1.605 | 1.842 | 1.809 | 1.656 | 1.792 | 1.762 | 1.867 | 1.818 | 1.815 |
| 21 | 1.792 | 1.789 | 1.597 | 1.825 | 1.276 | 1.866 | 1.728 | 1.795 | 1.515 | 1.802 | 1.574 | 1.824 |
| 22 | 1.815 | 1.850 | 1.753 | 1.763 | 1.807 | 1.647 | 1.575 | 1.792 | 1.747 | 1.782 | 1.800 | 1.729 |
| 23 | 1.873 | 1.782 | 1.825 | 1.746 | 1.358 | 1.767 | 1.744 | 1.800 | 1.812 | 1.831 | 1.838 | 1.702 |
| 24 | 1.866 | 1.811 | 1.852 | 1.826 | 1.714 | 1.777 | 1.834 | 1.817 | 1.556 | 1.791 | 1.778 | 1.775 |
| 25 | 1.715 | 1.724 | 1.862 | 1.722 | 1.823 | 1.826 | 1.876 | 1.834 | 1.728 | 1.786 | 1.722 | 1.559 |
| 26 | 1.634 | 1.777 | 1.751 | 1.793 | 1.803 | 1.784 | 1.675 | 1.802 | 1.828 | 1.785 | 1.780 | 1.649 |
| 27 | 1.787 | 1.741 | 1.815 | 1.872 | 1.766 | 1.795 | 1.827 | 1.744 | 1.751 | 1.803 | 1.833 | 1.813 |
| 28 | 1.865 | 1.805 | 1.821 | 1.707 | 1.760 | 1.832 | 1.706 | 1.769 | 1.672 | 1.647 | 1.536 | 1.590 |
| 29 | 1.861 | 1.842 | 1.776 | 1.806 | 1.445 | 1.836 | 1.674 | 1.827 | 1.766 | 1.782 | 1.764 | 1.795 |
| 30 | 1.853 | 1.801 | 1.459 | 1.765 | 1.685 | 1.861 | 1.679 | 1.713 | 1.799 | 1.775 | 1.550 | 1.827 |
| 31 | 1.846 | 1.590 | 1.670 | 1.662 | 1.730 | 1.771 | 1.797 | 1.834 | 1.847 | 1.755 | 1.722 | 1.819 |
| 32 | 1.843 | 1.516 | 1.823 | 1.662 | 1.820 | 1.833 | 1.656 | 1.833 | 1.841 | 1.798 | 1.822 | 1.797 |
| 33 | 1.790 | 1.848 | 1.855 | 1.049 | 1.296 | 1.840 | 1.439 | 1.851 | 1.806 | 1.795 | 1.873 | 1.493 |
| 34 | 1.849 | 1.794 | 1.803 | 1.827 | 1.577 | 1.734 | 1.638 | 1.878 | 1.729 | 1.812 | 1.844 | 1.763 |
| 35 | 1.793 | 1.443 | 1.768 | 1.821 | 1.473 | 1.744 | 1.343 | 1.830 | 1.856 | 1.778 | 1.725 | 1.805 |
| 36 | 1.782 | 1.618 | 1.847 | 1.818 | 1.893 | 1.850 | 1.822 | 1.796 | 1.837 | 1.804 | 1.840 | 1.806 |
| 37 | 1.781 | 1.808 | 1.894 | 1.855 | 1.507 | 1.808 | 1.237 | 1.825 | 1.796 | 1.753 | 1.454 | 1.762 |
| 38 | 1.855 | 1.767 | 1.858 | 1.827 | 1.673 | 1.644 | 1.734 | 1.864 | 1.839 | 1.726 | 1.862 | 1.264 |
| 39 | 1.764 | 1.804 | 1.856 | 1.901 | 1.500 | 1.787 | 1.838 | 1.853 | 1.807 | 1.731 | 1.745 | 1.545 |
| 40 | 1.821 | 1.793 | 1.768 | 1.804 | 1.479 | 1.806 | 1.839 | 1.757 | 1.826 | 1.764 | 1.751 | 1.790 |
| 41 | 1.862 | 1.759 | 1.816 | 1.835 | 1.442 | 1.821 | 1.696 | 1.871 | 1.793 | 1.726 | 1.849 | 1.478 |
| 42 | 1.784 | 1.764 | 1.801 | 1.755 | 1.678 | 1.824 | 1.659 | 1.849 | 1.791 | 1.857 | 1.644 | 1.673 |
| 43 | 1.826 | 1.846 | 1.824 | 1.725 | 1.805 | 1.816 | 1.590 | 1.842 | 1.744 | 1.820 | 1.462 | 1.708 |
| 44 | 1.391 | 1.797 | 1.788 | 1.846 | 1.804 | 1.726 | 1.781 | 1.837 | 1.616 | 1.747 | 1.657 | 1.790 |
| 45 | 1.820 | 1.803 | 1.462 | 1.816 | 1.814 | 1.802 | 1.831 | 1.612 | 1.655 | 1.801 | 1.805 | 1.656 |
| 46 | 1.717 | 1.776 | 1.782 | 1.829 | 1.791 | 1.678 | 1.831 | 1.786 | 1.414 | 1.785 | 1.808 | 1.636 |
| 47 | 1.747 | 1.815 | 1.862 | 1.884 | 1.628 | 1.754 | 1.760 | 1.815 | 1.862 | 1.827 | 1.828 | 1.739 |
| 48 | 1.838 | 1.822 | 1.728 | 1.903 | 1.767 | 1.804 | 1.701 | 1.821 | 1.765 | 1.861 | 1.735 | 1.649 |
| 49 | 1.841 | 1.773 | 1.717 | 1.712 | 1.815 |  | 1.859 | 1.784 | 1.858 | 1.667 | 1.794 | 1.808 |
| 50 | 1.831 |  | 1.720 | 1.892 | 1.862 |  | 1.757 | 1.721 | 1.837 | 1.783 | 1.791 | 1.778 |
| 51 |  |  | 1.823 | 1.852 | 1.757 |  | 1.714 | 1.704 | 1.814 |  | 1.728 | 1.825 |
| 52 |  |  |  | 1.664 |  |  | 1.626 | 1.748 | 1.759 |  | 1.724 | 1.841 |
| 53 |  |  |  | 1.861 |  |  | 1.793 | 1.734 |  |  | 1.762 | 1.837 |
| 54 |  |  |  | 1.543 |  |  | 1.801 | 1.777 |  |  | 1.892 | 1.810 |
| 55 |  |  |  | 1.799 |  |  | 1.644 | 1.732 |  |  | 1.569 | 1.905 |
| 56 |  |  |  | 1.720 |  |  | 1.576 | 1.721 |  |  |  | 1.826 |
| 57 |  |  |  | 1.815 |  |  | 1.486 | 1.598 |  |  |  | 1.776 |
| 58 |  |  |  | 1.818 |  |  | 1.301 | 1.790 |  |  |  | 1.796 |
| 59 |  |  |  | 1.765 |  |  | 1.319 | 1.687 |  |  |  | 1.842 |
| 60 |  |  |  | 1.624 |  |  | 1.338 | 1.661 |  |  |  | 1.830 |
| 61 |  |  |  | 1.762 |  |  |  | 1.773 |  |  |  | 1.600 |
| 62 |  |  |  | 1.745 |  |  |  | 1.737 |  |  |  | 1.771 |
| 63 |  |  |  | 1.795 |  |  |  | 1.590 |  |  |  | 1.766 |
| 64 |  |  |  | 1.551 |  |  |  | 1.821 |  |  |  | 1.601 |
| 65 |  |  |  | 1.747 |  |  |  | 1.870 |  |  |  | 1.499 |
| 66 |  |  |  | 1.845 |  |  |  | 1.738 |  |  |  | 1.474 |
| 67 |  |  |  | 1.714 |  |  |  | 1.616 |  |  |  | 1.741 |
| 68 |  |  |  | 1.803 |  |  |  | 1.771 |  |  |  | 1.791 |
| 69 |  |  |  | 1.796 |  |  |  | 1.839 |  |  |  | 1.805 |
| 70 |  |  |  | 1.740 |  |  |  | 1.659 |  |  |  | 1.833 |
| 71 |  |  |  | 1.836 |  |  |  | 1.736 |  |  |  | 1.757 |
| 72 |  |  |  | 1.894 |  |  |  | 1.738 |  |  |  | 1.726 |
| 73 |  |  |  | 1.767 |  |  |  | 1.673 |  |  |  | 1.800 |
| 74 |  |  |  | 1.770 |  |  |  |  |  |  |  | 1.749 |
| 75 |  |  |  | 1.293 |  |  |  |  |  |  |  | 1.738 |
| 76 |  |  |  | 1.633 |  |  |  |  |  |  |  | 1.594 |
| 77 |  |  |  | 1.794 |  |  |  |  |  |  |  | 1.698 |
| 78 |  |  |  | 1.818 |  |  |  |  |  |  |  | 1.687 |
| 79 |  |  |  | 1.841 |  |  |  |  |  |  |  |  |
| 80 |  |  |  | 1.843 |  |  |  |  |  |  |  |  |
| 81 |  |  |  | 1.768 |  |  |  |  |  |  |  |  |
| 82 |  |  |  | 1.867 |  |  |  |  |  |  |  |  |
| 83 |  |  |  | 1.847 |  |  |  |  |  |  |  |  |

Table S8. DFA (Detrended Fluctuation Analysis) scaling exponent γ values for the 700 experimental cell trajectories.

**Table S9**

| Cell number | Amoeba proteus | | | | Metamoeba leningradensis | | | | Amoeba borokensis | | | |
| --- | --- | --- | --- | --- | --- | --- | --- | --- | --- | --- | --- | --- |
|  | Sc1 | Sc2 | Sc3 | Sc4 | Sc1 | Sc2 | Sc3 | Sc4 | Sc1 | Sc2 | Sc3 | Sc4 |
| 1 | 0.582 | 0.412 | 0.344 | 0.615 | 0.325 | 0.660 | 0.531 | 0.350 | 0.410 | 0.474 | 0.310 | 0.659 |
| 2 | 0.570 | 0.670 | 0.577 | 0.505 | 0.477 | 0.624 | 0.507 | 0.426 | 0.312 | 0.389 | 0.519 | 0.351 |
| 3 | 0.318 | 0.470 | 0.472 | 0.482 | 0.291 | 0.329 | 0.506 | 0.507 | 0.500 | 0.349 | 0.563 | 0.443 |
| 4 | 0.496 | 0.346 | 0.421 | 0.411 | 0.529 | 0.510 | 0.694 | 0.293 | 0.512 | 0.393 | 0.351 | 0.410 |
| 5 | 0.577 | 0.579 | 0.568 | 0.350 | 0.280 | 0.603 | 0.499 | 0.610 | 0.435 | 0.456 | 0.497 | 0.536 |
| 6 | 0.435 | 0.435 | 0.483 | 0.383 | 0.462 | 0.323 | 0.454 | 0.448 | 0.352 | 0.373 | 0.627 | 0.372 |
| 7 | 0.493 | 0.411 | 0.472 | 0.464 | 0.699 | 0.591 | 0.357 | 0.539 | 0.509 | 0.467 | 0.424 | 0.509 |
| 8 | 0.582 | 0.467 | 0.570 | 0.592 | 0.566 | 0.563 | 0.421 | 0.500 | 0.393 | 0.633 | 0.621 | 0.417 |
| 9 | 0.725 | 0.491 | 0.249 | 0.330 | 0.402 | 0.496 | 0.400 | 0.508 | 0.628 | 0.519 | 0.620 | 0.520 |
| 10 | 0.515 | 0.637 | 0.615 | 0.615 | 0.322 | 0.413 | 0.560 | 0.439 | 0.421 | 0.545 | 0.397 | 0.347 |
| 11 | 0.320 | 0.637 | 0.618 | 0.493 | 0.476 | 0.501 | 0.491 | 0.514 | 0.380 | 0.417 | 0.376 | 0.555 |
| 12 | 0.492 | 0.631 | 0.420 | 0.331 | 0.510 | 0.640 | 0.585 | 0.275 | 0.580 | 0.450 | 0.314 | 0.584 |
| 13 | 0.479 | 0.326 | 0.488 | 0.505 | 0.397 | 0.628 | 0.274 | 0.667 | 0.520 | 0.554 | 0.522 | 0.680 |
| 14 | 0.591 | 0.435 | 0.542 | 0.382 | 0.443 | 0.314 | 0.396 | 0.431 | 0.434 | 0.447 | 0.390 | 0.561 |
| 15 | 0.480 | 0.353 | 0.456 | 0.546 | 0.444 | 0.433 | 0.401 | 0.358 | 0.607 | 0.461 | 0.337 | 0.460 |
| 16 | 0.559 | 0.500 | 0.517 | 0.460 | 0.330 | 0.322 | 0.439 | 0.375 | 0.567 | 0.538 | 0.493 | 0.432 |
| 17 | 0.498 | 0.523 | 0.641 | 0.525 | 0.315 | 0.590 | 0.288 | 0.566 | 0.460 | 0.462 | 0.419 | 0.583 |
| 18 | 0.618 | 0.530 | 0.499 | 0.450 | 0.362 | 0.578 | 0.500 | 0.560 | 0.515 | 0.616 | 0.335 | 0.446 |
| 19 | 0.480 | 0.476 | 0.375 | 0.434 | 0.492 | 0.339 | 0.523 | 0.654 | 0.458 | 0.453 | 0.473 | 0.437 |
| 20 | 0.565 | 0.440 | 0.504 | 0.563 | 0.369 | 0.616 | 0.385 | 0.330 | 0.411 | 0.372 | 0.544 | 0.517 |
| 21 | 0.573 | 0.505 | 0.409 | 0.564 | 0.470 | 0.567 | 0.401 | 0.395 | 0.701 | 0.290 | 0.435 | 0.599 |
| 22 | 0.361 | 0.581 | 0.346 | 0.397 | 0.507 | 0.384 | 0.538 | 0.407 | 0.566 | 0.375 | 0.496 | 0.492 |
| 23 | 0.448 | 0.507 | 0.451 | 0.381 | 0.430 | 0.346 | 0.572 | 0.534 | 0.471 | 0.541 | 0.450 | 0.558 |
| 24 | 0.605 | 0.530 | 0.403 | 0.526 | 0.631 | 0.368 | 0.278 | 0.584 | 0.388 | 0.485 | 0.444 | 0.552 |
| 25 | 0.348 | 0.491 | 0.545 | 0.344 | 0.302 | 0.405 | 0.597 | 0.602 | 0.536 | 0.531 | 0.587 | 0.443 |
| 26 | 0.427 | 0.401 | 0.455 | 0.481 | 0.368 | 0.474 | 0.567 | 0.464 | 0.563 | 0.429 | 0.472 | 0.530 |
| 27 | 0.644 | 0.328 | 0.413 | 0.409 | 0.390 | 0.437 | 0.445 | 0.507 | 0.456 | 0.249 | 0.689 | 0.542 |
| 28 | 0.495 | 0.504 | 0.412 | 0.566 | 0.448 | 0.509 | 0.560 | 0.452 | 0.557 | 0.284 | 0.548 | 0.403 |
| 29 | 0.494 | 0.526 | 0.414 | 0.657 | 0.466 | 0.645 | 0.420 | 0.417 | 0.452 | 0.560 | 0.449 | 0.447 |
| 30 | 0.564 | 0.293 | 0.514 | 0.446 | 0.445 | 0.481 | 0.348 | 0.446 | 0.313 | 0.380 | 0.508 | 0.645 |
| 31 | 0.430 | 0.465 | 0.473 | 0.399 | 0.500 | 0.464 | 0.410 | 0.400 | 0.395 | 0.361 | 0.301 | 0.411 |
| 32 | 0.506 | 0.352 | 0.442 | 0.257 | 0.477 | 0.539 | 0.523 | 0.345 | 0.544 | 0.649 | 0.449 | 0.345 |
| 33 | 0.420 | 0.530 | 0.329 | 0.534 | 0.506 | 0.635 | 0.802 | 0.522 | 0.578 | 0.386 | 0.452 | 0.540 |
| 34 | 0.557 | 0.509 | 0.569 | 0.410 | 0.481 | 0.714 | 0.510 | 0.565 | 0.431 | 0.449 | 0.400 | 0.560 |
| 35 | 0.454 | 0.572 | 0.415 | 0.417 | 0.545 | 0.446 | 0.389 | 0.351 | 0.486 | 0.587 | 0.466 | 0.619 |
| 36 | 0.451 | 0.542 | 0.471 | 0.417 | 0.547 | 0.410 | 0.368 | 0.454 | 0.389 | 0.757 | 0.421 | 0.393 |
| 37 | 0.584 | 0.623 | 0.370 | 0.476 | 0.470 | 0.401 | 0.487 | 0.413 | 0.382 | 0.603 | 0.401 | 0.494 |
| 38 | 0.365 | 0.495 | 0.549 | 0.411 | 0.561 | 0.486 | 0.393 | 0.488 | 0.348 | 0.495 | 0.431 | 0.371 |
| 39 | 0.503 | 0.435 | 0.591 | 0.513 | 0.710 | 0.570 | 0.535 | 0.373 | 0.500 | 0.619 | 0.529 | 0.514 |
| 40 | 0.514 | 0.561 | 0.505 | 0.445 | 0.454 | 0.339 | 0.404 | 0.320 | 0.337 | 0.500 | 0.385 | 0.490 |
| 41 | 0.702 | 0.413 | 0.502 | 0.441 | 0.507 | 0.464 | 0.646 | 0.438 | 0.453 | 0.400 | 0.530 | 0.669 |
| 42 | 0.456 | 0.405 | 0.366 | 0.579 | 0.534 | 0.646 | 0.589 | 0.626 | 0.525 | 0.654 | 0.471 | 0.416 |
| 43 | 0.459 | 0.509 | 0.531 | 0.556 | 0.249 | 0.461 | 0.511 | 0.472 | 0.375 | 0.433 | 0.571 | 0.484 |
| 44 | 0.483 | 0.516 | 0.247 | 0.391 | 0.446 | 0.483 | 0.494 | 0.465 | 0.393 | 0.378 | 0.549 | 0.394 |
| 45 | 0.542 | 0.411 | 0.480 | 0.762 | 0.442 | 0.337 | 0.516 | 0.350 | 0.306 | 0.507 | 0.655 | 0.415 |
| 46 | 0.488 | 0.460 | 0.489 | 0.321 | 0.595 | 0.563 | 0.419 | 0.521 | 0.447 | 0.486 | 0.273 | 0.412 |
| 47 | 0.642 | 0.461 | 0.458 | 0.316 | 0.471 | 0.571 | 0.555 | 0.592 | 0.396 | 0.511 | 0.378 | 0.571 |
| 48 | 0.525 | 0.425 | 0.495 | 0.521 | 0.311 | 0.517 | 0.516 | 0.577 | 0.537 | 0.473 | 0.660 | 0.476 |
| 49 | 0.525 | 0.352 | 0.636 | 0.463 | 0.467 |  | 0.508 | 0.640 | 0.597 | 0.349 | 0.424 | 0.633 |
| 50 | 0.462 |  | 0.667 | 0.441 | 0.462 |  | 0.539 | 0.706 | 0.515 | 0.526 | 0.418 | 0.431 |
| 51 |  |  | 0.326 | 0.488 | 0.427 |  | 0.488 | 0.478 | 0.472 |  | 0.341 | 0.447 |
| 52 |  |  |  | 0.481 |  |  | 0.407 | 0.509 | 0.401 |  | 0.479 | 0.373 |
| 53 |  |  |  | 0.480 |  |  | 0.586 | 0.646 |  |  | 0.677 | 0.445 |
| 54 |  |  |  | 0.377 |  |  | 0.527 | 0.243 |  |  | 0.374 | 0.533 |
| 55 |  |  |  | 0.308 |  |  | 0.626 | 0.545 |  |  | 0.403 | 0.366 |
| 56 |  |  |  | 0.355 |  |  | 0.509 | 0.408 |  |  |  | 0.361 |
| 57 |  |  |  | 0.491 |  |  | 0.396 | 0.458 |  |  |  | 0.633 |
| 58 |  |  |  | 0.556 |  |  | 0.570 | 0.603 |  |  |  | 0.460 |
| 59 |  |  |  | 0.695 |  |  | 0.469 | 0.445 |  |  |  | 0.369 |
| 60 |  |  |  | 0.560 |  |  | 0.351 | 0.333 |  |  |  | 0.493 |
| 61 |  |  |  | 0.426 |  |  |  | 0.562 |  |  |  | 0.351 |
| 62 |  |  |  | 0.585 |  |  |  | 0.464 |  |  |  | 0.628 |
| 63 |  |  |  | 0.475 |  |  |  | 0.562 |  |  |  | 0.604 |
| 64 |  |  |  | 0.603 |  |  |  | 0.564 |  |  |  | 0.544 |
| 65 |  |  |  | 0.449 |  |  |  | 0.361 |  |  |  | 0.537 |
| 66 |  |  |  | 0.349 |  |  |  | 0.527 |  |  |  | 0.417 |
| 67 |  |  |  | 0.638 |  |  |  | 0.512 |  |  |  | 0.586 |
| 68 |  |  |  | 0.578 |  |  |  | 0.474 |  |  |  | 0.384 |
| 69 |  |  |  | 0.478 |  |  |  | 0.394 |  |  |  | 0.565 |
| 70 |  |  |  | 0.469 |  |  |  | 0.369 |  |  |  | 0.325 |
| 71 |  |  |  | 0.331 |  |  |  | 0.480 |  |  |  | 0.568 |
| 72 |  |  |  | 0.470 |  |  |  | 0.551 |  |  |  | 0.320 |
| 73 |  |  |  | 0.510 |  |  |  | 0.559 |  |  |  | 0.628 |
| 74 |  |  |  | 0.510 |  |  |  |  |  |  |  | 0.472 |
| 75 |  |  |  | 0.659 |  |  |  |  |  |  |  | 0.632 |
| 76 |  |  |  | 0.599 |  |  |  |  |  |  |  | 0.414 |
| 77 |  |  |  | 0.323 |  |  |  |  |  |  |  | 0.426 |
| 78 |  |  |  | 0.503 |  |  |  |  |  |  |  | 0.492 |
| 79 |  |  |  | 0.442 |  |  |  |  |  |  |  |  |
| 80 |  |  |  | 0.549 |  |  |  |  |  |  |  |  |
| 81 |  |  |  | 0.431 |  |  |  |  |  |  |  |  |
| 82 |  |  |  | 0.511 |  |  |  |  |  |  |  |  |
| 83 |  |  |  | 0.550 |  |  |  |  |  |  |  |  |

Table S9. DFA (Detrended Fluctuation Analysis) scaling exponent γ values for the 700 shuffled cell trajectories.

**Table S10**

| Species | Scenario | Intensity of Response | Directionality Ratio | Average Speed |
| --- | --- | --- | --- | --- |
| Amoeba Proteus | Sc1-Sc2 | 0.006 | 10^−8^ | 0.002 |
|  | Sc1-Sc3 | 0.959 | 0.080 | 10^−4^ |
|  | Sc1-Sc4 | 0.155 | 0.084 | 10^−12^ |
|  | Sc2-Sc3 | 10^−4^ | 10^−7^ | 0.544 |
|  | Sc2-Sc4 | 10^−8^ | 10^−7^ | 0.001 |
|  | Sc3-Sc4 | 0.100 | 0.865 | 0.005 |
|  | All | 10^−6^ | 10^−9^ | 10^−10^ |
| Metamoeba leningradensis | Sc1-Sc2 | 10^−8^ | 10^−8^ | 10^−4^ |
|  | Sc1-Sc3 | 0.098 | 0.734 | 0.004 |
|  | Sc1-Sc4 | 10^−13^ | 10^−6^ | 10^−13^ |
|  | Sc2-Sc3 | 10^−5^ | 10^−9^ | 0.899 |
|  | Sc2-Sc4 | 0.010 | 0.050 | 10^−8^ |
|  | Sc3-Sc4 | 10^−11^ | 10^−7^ | 10^−8^ |
|  | All | 10^−16^ | 10^−12^ | 10^−15^ |
| Amoeba borokensis | Sc1-Sc2 | 10^−4^ | 10^−5^ | 0.160 |
|  | Sc1-Sc3 | 0.884 | 0.918 | 0.326 |
|  | Sc1-Sc4 | 0.099 | 0.862 | 10^−4^ |
|  | Sc2-Sc3 | 0.003 | 10^−4^ | 0.982 |
|  | Sc2-Sc4 | 0.029 | 10^−4^ | 0.119 |
|  | Sc3-Sc4 | 0.133 | 0.659 | 0.230 |
|  | All | 0.002 | 10^−5^ | 0.022 |
| Amoeba proteus  Vs.  Metamoeba leningradensis | Sc1-Sc2 | 0.263 | 10^−7^ | 10^−8^ |
|  | Sc1-Sc3 | 0.006 | 0.318 | 10^−7^ |
|  | Sc1-Sc4 | 10^−4^ | 10^−5^ | 0.417 |
|  | Sc2-Sc3 | 10^−8^ | 10^−10^ | 0.010 |
|  | Sc2-Sc4 | 0.573 | 0.002 | 10^−4^ |
|  | Sc3-Sc4 | 10^−4^ | 0.003 | 10^−5^ |
|  | All | 0.293 | 0.793 | 0.065 |
| Amoeba proteus  Vs.  Amoeba borokensis | Sc1-Sc2 | 0.537 | 10^−5^ | 10^−11^ |
|  | Sc1-Sc3 | 0.002 | 0.895 | 10^−8^ |
|  | Sc1-Sc4 | 0.009 | 0.559 | 10^−12^ |
|  | Sc2-Sc3 | 10^−8^ | 10^−7^ | 10^−4^ |
|  | Sc2-Sc4 | 10^−9^ | 10^−7^ | 10^−5^ |
|  | Sc3-Sc4 | 0.004 | 0.106 | 10^−4^ |
|  | All | 10^−10^ | 0.032 | 10^−17^ |
| Metamoeba leningradensis  Vs.  Amoeba borokensis | Sc1-Sc2 | 10^−5^ | 10^−6^ | 0.064 |
|  | Sc1-Sc3 | 0.383 | 0.515 | 0.131 |
|  | Sc1-Sc4 | 0.005 | 0.228 | 0.001 |
|  | Sc2-Sc3 | 10^−6^ | 10^−7^ | 0.085 |
|  | Sc2-Sc4 | 10^−5^ | 10^−7^ | 0.375 |
|  | Sc3-Sc4 | 0.529 | 0.117 | 0.401 |
|  | All | 10^−5^ | 0.067 | 10^−7^ |

Table S10. Kruskal-Wallis and Wilcoxon analyses to assess the variability in kinetic properties.

**SI References**

1. W. Korohoda, M. Mycielska, E. Janda, Z. Madeja, Immediate and long-term galvanotactic responses of Amoeba proteus to dc electric fields. *Cell Motil. Cytoskeleton* **45**, 10–26 (2000).

2. A. Goodkov, A. Yudin, Y. Podlipaeva, Collection of the proteus-type amoebae at the institute of cytology, russian academy of sciences. I. history, goals and research fields. *Protistology* **8**, 71–75 (2014).

3. D. M. Prescott, R. F. Carrier, “Experimental Procedures and Cultural Methods for Euplotes eurystomus and Amoeba proteus” in *Methods in Cell Biology*, D. M. Prescott, Ed. (Academic Press, 1964), pp. 85–95.

4. M. R. Green, J. Sambrook, *Molecular Cloning: A Laboratory Manual*, 4th ed. (Cold Spring Harbor Laboratory Press, 2012).

5. J.-Y. Tinevez, *et al.*, TrackMate: An open and extensible platform for single-particle tracking. *Methods* **115**, 80–90 (2017).

6. O. Hilsenbeck, *et al.*, Software tools for single-cell tracking and quantification of cellular and molecular properties. *Nat. Biotechnol.* **34**, 703–706 (2016).

7. J. W. Gibbs, *Elementary principles in statistical mechanics: developed with especial reference to the rational foundations of thermodynamics* (Charles Scribner’s sons, 1902).

8. A. Einstein, Zum gegenwärtigen Stand des Strahlungsproblems. *Phys. Z.* **10**, 185–193 (1909).

9. P. Ch. Ivanov, *et al.*, Multifractality in human heartbeat dynamics. *Nature* **399**, 461–465 (1999).

10. P. Ch. Ivanov, *et al.*, From 1/f noise to multifractal cascades in heartbeat dynamics. *Chaos Interdiscip. J. Nonlinear Sci.* **11**, 641–652 (2001).

11. G. M. Viswanathan, *et al.*, Lévy flight search patterns of wandering albatrosses. *Nature* **381**, 413–415 (1996).

12. A. Einstein, Über die von der molekularkinetischen Theorie der Wärme geforderte Bewegung von in ruhenden Flüssigkeiten suspendierten Teilchen. *Ann. Phys.* **322**, 549–560 (1905).

13. Z. Long, *et al.*, Microfluidic chemostat for measuring single cell dynamics in bacteria. *Lab Chip* **13**, 947–954 (2013).

14. R. Gorelik, A. Gautreau, Quantitative and unbiased analysis of directional persistence in cell migration. *Nature Protocols* **9**, 1931–1943 (2014).

15. G. M. Viswanathan, E. P. Raposo, M. G. E. da Luz, Lévy flights and superdiffusion in the context of biological encounters and random searches. *Phys. Life Rev.* **5**, 133–150 (2008).

16. C.-K. Peng, *et al.*, Mosaic organization of DNA nucleotides. *Phys Rev E* **49**, 1685–1689 (1994).

17. A. L. Goldberger, *et al.*, Fractal dynamics in physiology: Alterations with disease and aging. *Proceedings of the National Academy of Sciences* **99**, 2466–2472 (2002).

18. S. M. Pincus, I. M. Gladstone, R. A. Ehrenkranz, A regularity statistic for medical data analysis. *J. Clin. Monit.* **7**, 335–345 (1991).

19. S. M. Pincus, Approximate entropy as a measure of system complexity. *Proceedings of the National Academy of Sciences* **88**, 2297–2301 (1991).

20. L. Cao, Practical method for determining the minimum embedding dimension of a scalar time series. *Physica D: Nonlinear Phenomena* **110**, 43–50 (1997).
